# Trends in global burden of diseases attributable to lead exposure in 204 countries and territories from 1990 to 2019

**DOI:** 10.3389/fpubh.2022.1036398

**Published:** 2022-11-23

**Authors:** Nan Zhou, Yue Huang, Mingma Li, Lu Zhou, Hui Jin

**Affiliations:** ^1^Key Laboratory of Environmental Medicine and Engineering of Ministry of Education, Department of Epidemiology and Statistics, School of Public Health, Southeast University, Nanjing, China; ^2^Jiangsu Provincial Center for Disease Control and Prevention, Nanjing, China

**Keywords:** lead exposure, global burden, cardiovascular diseases, chronic kidney disease, idiopathic developmental intellectual disability

## Abstract

**Background:**

Lead hazards are ubiquitous in the environment, and lead exposure has been proved to damage human health. Nevertheless, there is limited data on the global burden of diseases attributable to lead exposure. In this study, we evaluated the temporal-spatial trend of disease burden caused by lead exposure in 204 countries and territories from 1990 to 2019.

**Methods:**

Based on Global Burden of Disease (GBD) Study 2019, deaths, disability-adjusted life years (DALYs), age-standardized mortality rate (ASMR) and DALYs rate (ASDR) were estimated by region, country, sex and age. The estimated annual percentage change (EAPC) was calculated to assess the temporal trends of ASMR and ASDR between 1990 and 2019.

**Results:**

Global deaths increased from 0.53 (95% UI: 0.31, 0.77) to 0.90 (95% UI: 0.55, 1.29) million, and the number of DALYs increased from 16.02 (95% UI: 10.32, 22.17) to 21.68 (95% UI: 13.81, 30.30) million between 1990 and 2019. China, India and Bangladesh were top three countries with the largest number of deaths and DALYs in 2019. The ASMR (per 100,000 population) decreased from 14.47 (95% UI: 8.40, 21.43) to 11.48 (95% UI: 7.00, 16.49) with EAPC of −0.75 (95% UI: −0.87, −0.64), and the ASDR (per 100,000 population) decreased from 378.01 (95% UI: 240.55, 524.18) to 267.52 (95% UI: 170.57, 373.44) with EAPC of −1.19 (95% UI: −1.32, −1.07). Most of disease burden of lead exposure occurred in the men and elderly population. Stroke and ischemic heart disease were two key sources of disease burden of lead exposure. Also, a negative association between sociodemographic index (SDI) and disease burden of lead exposure was observed.

**Conclusions:**

Lead exposure poses a significant disease burden globally, and is still a great threat to public health. Primary prevention measures of reducing lead exposure in the environment are essential.

## Introduction

Lead is a persistent metal and has been widely used in various industrial products like cosmetics, medicines, paint, and petrol (1). Lead contamination was observed in different environmental media like air, water, dust, and soil (2). Humans can be exposed to lead by inhaling contaminated air, ingesting contaminated food and water, or direct contact (2). Although lead exposure fell substantially with the expanding use of unleaded paint and petrol, humans still live in a world containing numerous leaded products, such as batteries, water pipes, and electronic waste (3), and lead continues to be a widespread environmental contaminant.

There is almost no physiological function for lead. When ingested or inhaled, lead can enter the blood and finally accumulate in the bones like many other “bone-seeking” elements. The half-life of bone lead can be up to 30 years (4). In the body, lead can increase oxidative stress and then result in adverse effects on human health (5). For example, lead exposure can damage the nervous system because the brain is the most sensitive organ to lead toxicity (6) and can reduce children's intelligence quotient (IQ) and result in multiple mental disorders, such as Alzheimer's disease, idiopathic developmental intellectual disability, Parkinson's disease, and attention-deficit hyperactivity disorder (7). Higher blood lead levels also indicate a higher risk of systolic left ventricular dysfunction and can increase circulatory mortality (8, 9). Lead has met the burden of proof to be considered a coronary risk factor (10). As the kidney excretes toxicants by urine, it is faced with higher lead poisoning. The higher concentrations of lead in blood have an adverse effect on intercellular junctions and alter the biochemical characteristics of the renal proximal tubule cells, causing renal injury and reducing the glomerular filtration rate (GFR) (11). The association between lead exposure and the development of kidney disease has been discussed (12). Besides, lead can injure other systems like reproductive and hematopoietic systems (5, 13, 14).

Overall, lead exposure is still a significant public health risk, but current studies on disease burden attributable to lead exposure are regional and limited. Although the released results of the Global Burden of Disease (GBD) Study 2019 provide us an opportunity to obtain the overall perspective of disease burden due to lead exposure, there is only one GBD study focusing on one cause (15). The global temporal-spatial trend of disease burden due to lead exposure is unknown. Thus, in this study, we analyzed the data from GBD 2019 in order to present valuable information for the global prevention and control of diseases related to lead exposure.

## Materials and methods

### Overview

In GBD 2019, the disease burden of 369 diseases and injuries in 204 countries and territories from 1990 to 2019 were obtained, including the number of deaths, disability-adjusted life years (DALY, a measure of overall disease burden, expressed as the number of years lost due to ill-health, disability, or early death), the age-standardized mortality rate (ASMR, a weighted average of the age-specific mortality rates per 100,000 persons), and age-standardized DALY rate (ASDR, adjusted DALY for differences in the age distribution of the population and expressed in per 100,000 population) (16). All causes were classified into 4 levels (Levels 1–4), and locations were clustered into 21 regions and 7 super-regions according to epidemiological similarities and geographical contiguity (17). In addition, five groups were recognized according to the sociodemographic index (SDI), which is a composite average of the rankings of the income per capita, average educational attainment, and fertility rates of areas in the GBD study, ranging from 0 (worst) to 1 (best) (18). Demographic characteristics in GBD 2019 were sex and age. Additionally, 87 risk factors were grouped into 4 levels. All data in GBD 2019 can be retrieved from the GBD Results Tool and be used to analyze the disease burden attributable to a specific cause or risk factor.

### Estimation of the disease burden attributable to lead exposure

In GBD 2019, lead exposure is defined in two ways. Acute lead exposure is measured as blood lead level (μg/dl), which is related to IQ loss in children. Chronic lead exposure is measured as bone lead level (μg/g) and is associated with increased systolic blood pressure and cardiovascular diseases. Data processing, exposure modeling, and estimating attributable burdens were described in detail (18). Briefly, the input data for lead exposure were from blood lead levels in the literature and a few blood lead surveys covering 552 different studies from 84 countries. The spatiotemporal Gaussian process regression (ST-GPR) was updated as the exposure modeling methodology to produce estimates of the mean and standard deviation of blood lead for all age groups, for both sexes, and all GBD locations from 1970 to 2019, and the theoretical minimum-risk exposure level (TMREL) was estimated at 2.0 μg/dl. Blood lead relative risks were taken from GBD 2017 using a reanalysis of a 2005 pooled study (19). The bone lead relative risks were taken from a 2008 meta-analysis (20). Then, the standard GBD population attributable fraction (PAF) equation was used to calculate PAFs using exposure estimates and relative risks. A Bayesian, regularized, trimmed meta-regression was used to estimate the excess mortality (15). Estimates of attributable burden as DALYs for risk-outcome pairs were generated by a mathematical model (18). In GBD 2019, blood lead level is paired with idiopathic developmental intellectual disability as modeled through the impact of blood lead levels on IQ in children, and bone lead level is paired with systolic blood pressure, and subsequently with all cardiovascular outcomes to which systolic blood pressure is paired. As a result, the disease burden due to lead exposure was all from non-communicable diseases (Level 1 cause). Cardiovascular diseases, mental disorders (only available in DALY and ASDR), and diabetes and kidney diseases were the Level 2 causes. The causes of Level 3 were as follows: rheumatic heart disease, ischemic heart disease, stroke, hypertensive heart disease, non-rheumatic valvular heart disease, cardiomyopathy and myocarditis, atrial fibrillation and flutter, aortic aneurysm, peripheral artery disease, endocarditis, other cardiovascular and circulatory diseases, idiopathic developmental intellectual disability (only available in DALY and ASDR), and chronic kidney disease.

### Statistical analysis

The 95% uncertainty intervals (95% UIs) of estimations of the disease burden attributable to lead exposure were computed according to the 2.5th and 97.5th centiles of 1,000 random draws of the uncertainty distribution (15). A regression line was fitted to calculate the estimated annual percentage change (EAPC) to assess the temporal trends of ASMR and ASDR due to lead exposure over the time interval between 1990 and 2019. The regression model is ln(ASMR or ASDR) = *α* + *βx* + *ε*, where *x* is the calendar year and β stands for the annual change. The EAPC was calculated as 100 × (exp(*β*)-1) and its 95% confidence interval (CI) can also be obtained from this model (21). If the upper limit of 95% CI was lower than zero, it was regarded as a decrease in the age-standardized rate. If the lower limit of 95% CI was higher than zero, it was regarded as an increase in the age-standardized rate. Otherwise, the age-standardized rate was considered to be stable. The associations between SDI and age-standardized rate and EAPC of age-standardized rate were described by Spearman rank correlation. Data statistics were conducted using the R program (Version 4.1.1, R Foundation for Statistical Computing, Vienna, Austria). A *p*-value of **<** 0.05 was deemed statistically significant.

## Results

### Global burden of diseases attributable to lead exposure

From 1990 to 2019, the global number of deaths attributable to lead exposure increased from 0.53 (95% UI: 0.31, 0.77) to 0.90 (95% UI: 0.55, 1.29) million, while the ASMR (per 100,000 population) decreased from 14.47 (95% UI: 8.40, 21.43) to 11.48 (95% UI: 7.00, 16.49) with an EAPC of −0.75 (95% UI: −0.87, −0.64). The number of DALYs (in millions) increased from 16.02 (95% UI: 10.32, 22.17) to 21.68 (95% UI: 13.81, 30.30). However, the ASDR (per 100,000 population) decreased from 378.01 (95% UI: 240.55, 524.18) to 267.52 (95% UI: 170.57, 373.44) with an EAPC of −1.19 (95% UI: −1.32, −1.07) (Table 1).

**Table 1:** Disease burden attributable to lead exposure from 1990 to 2019.

	**Deaths** **No**. × **10**^**3**^ **(95% UI)**		**ASMR per 10**^**5**^ **(95% UI)**			**DALYs** **No**. × **10**^**4**^ **(95% UI)**		**ASDR per 10**^**5**^ **(95% UI)**		
	**1990**	**2019**	**1990**	**2019**	**EAPC**	**1990**	**2019**	**1990**	**2019**	**EAPC**
Overall	529.94 (312.63, 772.03)	901.72 (550.91, 1,288.85)	14.47 (8.40, 21.43)	11.48 (7.00, 16.49)	−0.75 (−0.87, −0.64)	1,602.50 (1,032.09, 2,216.83)	2,167.64 (1,381.29, 3,029.77)	378.01 (240.55, 524.18)	267.52 (170.57, 373.44)	−1.19 (−1.32, −1.07)
**Cardiovascular diseases**										
Rheumatic heart disease	9.39 (5.10, 16.10)	7.69 (4.09, 13.50)	0.23 (0.12, 0.41)	0.10 (0.05, 0.17)	−3.03 (−3.14, −2.91)	32.02 (16.89, 54.36)	21.35 (10.68, 37.27)	7.18 (3.87, 12.16)	2.58 (1.28, 4.55)	−3.53 (−3.67, −3.39)
Ischemic heart disease	220.99 (124.89, 334.74)	413.04 (242.84, 615.75)	6.12 (3.36, 9.46)	5.26 (3.08, 7.88)	−0.47 (−0.55, −0.39)	540.86 (317.83, 795.06)	836.87 (489.64, 1,244.92)	134.35 (78.06, 199.09)	102.26 (59.74, 152.87)	−0.88 (−0.99, −0.78)
Stroke	207.41 (124.08, 304.80)	305.27 (182.80, 435.67)	5.53 (3.28, 8.22)	3.85 (2.30, 5.50)	−1.34 (−1.57, −1.10)	543.67 (325.34, 795.18)	673.88 (391.22, 981.56)	133.36 (80.09, 195.09)	81.97 (47.86, 119.11)	−1.34 (−1.95, −1.52)
Hypertensive heart disease	57.00 (22.33, 125.06)	97.49 (30.51, 225.24)	1.61 (0.57, 3.54)	1.27 (0.39, 2.96)	−0.78 (−0.95, −0.62)	130.84 (59.04, 267.63)	176.95 (66.37, 384.55)	33.36 (14.33, 69.08)	22.04 (8.09, 47.48)	−1.41 (−1.56, −1.27)
Non-rheumatic valvular heart disease	0.94 (0.41, 1.59)	1.92 (0.83, 3.40)	0.03 (0.01, 0.05)	0.03 (0.01, 0.05)	−0.36 (−0.50, −0.23)	2.05 (0.89, 3.47)	3.19 (1.47, 5.43)	0.52 (0.23, 0.88)	0.40 (0.18, 0.68)	−1.02 (−1.11, −0.93)
Cardiomyopathy and myocarditis	2.56 (1.05, 4.52)	3.48 (1.45, 6.35)	0.08 (0.03, 0.14)	0.05 (0.019, 0.08)	−2.00 (−2.13, −1.86)	6.21 (2.57, 10.81)	7.45 (2.94, 13.66)	1.57 (0.65, 2.70)	0.92 (0.36, 1.68)	−2.01 (−2.16, −1.86)
Atrial fibrillation and flutter	2.35 (1.21, 3.77)	7.10 (4.00, 11.09)	0.08 (0.04, 0.13)	0.10 (0.05, 0.15)	0.66 (0.57, 0.75)	10.22 (5.31, 16.48)	22.75 (12.45, 36.30)	2.75 (1.44, 4.45)	2.85 (1.56, 4.57)	0.17 (0.09, 0.25)
Aortic aneurysm	1.99 (0.90, 3.39)	3.47 (1.74, 3.47)	0.05 (0.02, 0.09)	0.04 (0.02, −0.07)	−0.95 (−1.10, −0.80)	4.67 (2.08, 8.10)	7.01 (3.48, 11.51)	1.15 (0.51, 2.00)	0.85 (0.42, 1.40)	−1.26 (−1.41, −1.11)
Peripheral artery disease	0.30 (0.10, 0.68)	0.78 (0.30, 1.68)	0.01 (0.00, 0.02)	0.01 (0.00, 0.02)	0.15 (0.004, 0.30)	0.98 (0.39, 1.91)	2.00 (0.86, 3.77)	0.27 (0.11, 0.54)	0.25 (0.11, 0.48)	−0.32 (−0.44, −0.20)
Endocarditis	0.61 (0.31, 1.02)	1.19 (0.55, 2.09)	0.02 (0.00, 0.03)	0.02 (0.00, 0.03)	−0.06 (−0.35, 0.22)	1.96 (0.96, 3.34)	2.79 (1.26, 5.05)	0.45 (0.22, 0.74)	0.34 (0.15, 0.62)	−1.03 (−1.27, −0.80)
Other cardiovascular and circulatory diseases	4.90 (2.70, 7.84)	7.33 (4.30, 10.81)	0.13 (0.07, 0.21)	0.09 (0.05, 0.14)	−1.23 (−1.31, −1.14)	14.96 (8.04, 24.31)	19.24 (10.42, 30.37)	3.56 (1.94, 5.75)	2.33 (1.26, 3.69)	−1.48 (−1.59, −1.37)
**Mental disorders**										
Idiopathic developmental intellectual disability						251.77 (114.27, 435.40)	271.63 (120.99, 483.47)	44.18 (19.98, 76.60)	35.70 (15.89, 63.60)	−0.78 (−0.90, −0.66)
**Diabetes and kidney diseases**										
Chronic kidney disease	21.39 (12.89, 30.89)	52.94 (31.64, 76.23)	0.59 (0.35, 0.85)	0.68 (0.40, 0.98)	0.60 (0.39, 0.81)	62.28 (37.33, 90.76)	122.52 (70.79, 181.80)	15.29 (9.19, 22.14)	15.02 (8.68, 22.26)	0.08 (−0.14, 0.29)

DALY, disability-adjusted life year; ASMR, age-standardized mortality rate; ASDR, age-standardized DALY rate; EAPC, estimated annual percentage change.

In 1990, cardiovascular diseases accounted for 95.97% of total deaths, and diabetes and kidney diseases accounted for 4.03%. For each cause of GBD Level 3, the five leading causes of death in 1990 were ischemic heart disease, stroke, hypertensive heart disease, chronic kidney disease, and rheumatic heart disease, accounting for 97.42% of total deaths. In 2019, cardiovascular diseases took up approximately 94.13% of total deaths, and diabetes and kidney diseases accounted for 5.87%. In 2019, the top five leading causes of GBD Level 3 remained unchanged compared to 1990, accounting for 97.20% of total deaths. In addition, ASMR in most GBD Level 3 causes had a decreasing trend, except for atrial fibrillation and flutter, peripheral artery disease, and chronic kidney disease, with an EAPC of 0.66 (95% CI: 0.57, 0.75), 0.15 (95% CI: 0.004, 0.30), and 0.60 (95% CI: 0.39, 0.81), respectively. Rheumatic heart disease, cardiomyopathy and myocarditis, and stroke had a substantially decreasing trend with the EAPC of −3.03 (95% CI: −3.14, −2.91), −2.00 (95% CI: −2.13, −1.86), and −1.34 (95% CI: −1.57, −1.10), respectively (Table 1).

For DALYs, cardiovascular diseases, mental disorders and diabetes, and kidney diseases accounted for 80.40, 15.71, and 3.89% of total DALYs in 1990, respectively. For each cause of GBD Level 3, stroke, ischemic heart disease, idiopathic developmental intellectual disability, hypertensive heart disease, and chronic kidney disease were the five leading causes, accounting for 95.44% of total DALYs in 1990. In 2019, cardiovascular diseases, mental disorders and diabetes, and kidney diseases accounted for 81.82, 12.53, and 5.65% of total DALYs, respectively. The five leading causes of GBD Level 3 were ischemic heart disease, stroke, idiopathic developmental intellectual disability, hypertensive heart disease, and chronic kidney disease, accounting for 96.04% of total DALYs. Except for atrial fibrillation and flutter with an EAPC of 0.17 (95% CI: 0.09, 0.25), ASDR in most causes of GBD Level 3 presented a downward trend. Rheumatic heart disease, cardiomyopathy and myocarditis, and stroke showed a significant decreasing trend with EAPC of −3.53 (95%CI: −3.67, −3.39), −2.01 (95%CI: −2.16, −1.86), and −1.34 (95%CI: −1.95, −1.52), respectively (Table 1).

### Global burden of diseases attributable to lead exposure by region

**Figure 1:**
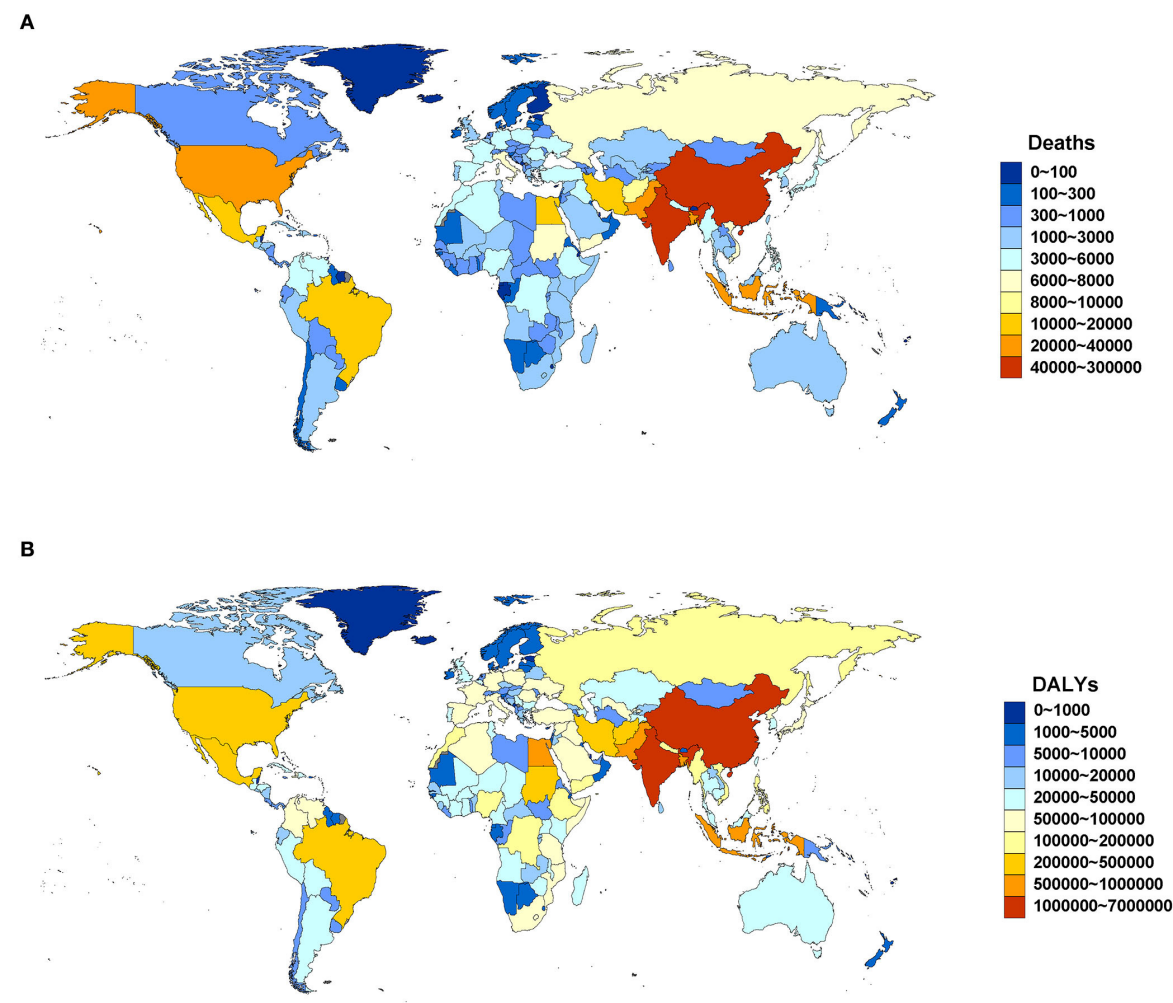
Deaths **(A)** and DALYs **(B)** attributable to lead exposure by country and territory in 2019. DALYs, disability-adjusted life years.

Regionally, South Asia (288.27, 95% UI: 200.62, 382.88) and East Asia (287.10, 95% UI: 179.33, 408.08) were the two GBD regions with the largest number (10^3^) of lead exposure-related deaths in 2019. The top five countries and territories by deaths were China (282.20, 95% UI: 177.00, 400.75), India (232.51, 95% UI: 161.34, 310.53), Bangladesh (30.78, 95% UI: 20.28, 42.15), Indonesia (27.40, 95% UI: 13.71, 43.05), and Pakistan (21.17, 95% UI: 12.74, 31.15) (Figure 1A; Supplementary Table 1). South Asia (843.94, 95%UI: 602.54, 1105.69) and East Asia (577.68, 95%UI: 359.48, 812.20) were also two GBD regions with the largest number (10^4^) of DALYs in 2019. The top five countries and territories of DALYs were India (696.40, 95% UI: 495.10, 909.70), China (565.79, 95% UI: 353.74, 794.85), Indonesia (71.15, 95% UI: 34.98, 115.67), Bangladesh (69.04, 95% UI: 46.06, 94.37), and Pakistan (67.87, 95% UI: 41.16, 98.20) (Figure 1B; Supplementary Table 2).

**Figure 2:**
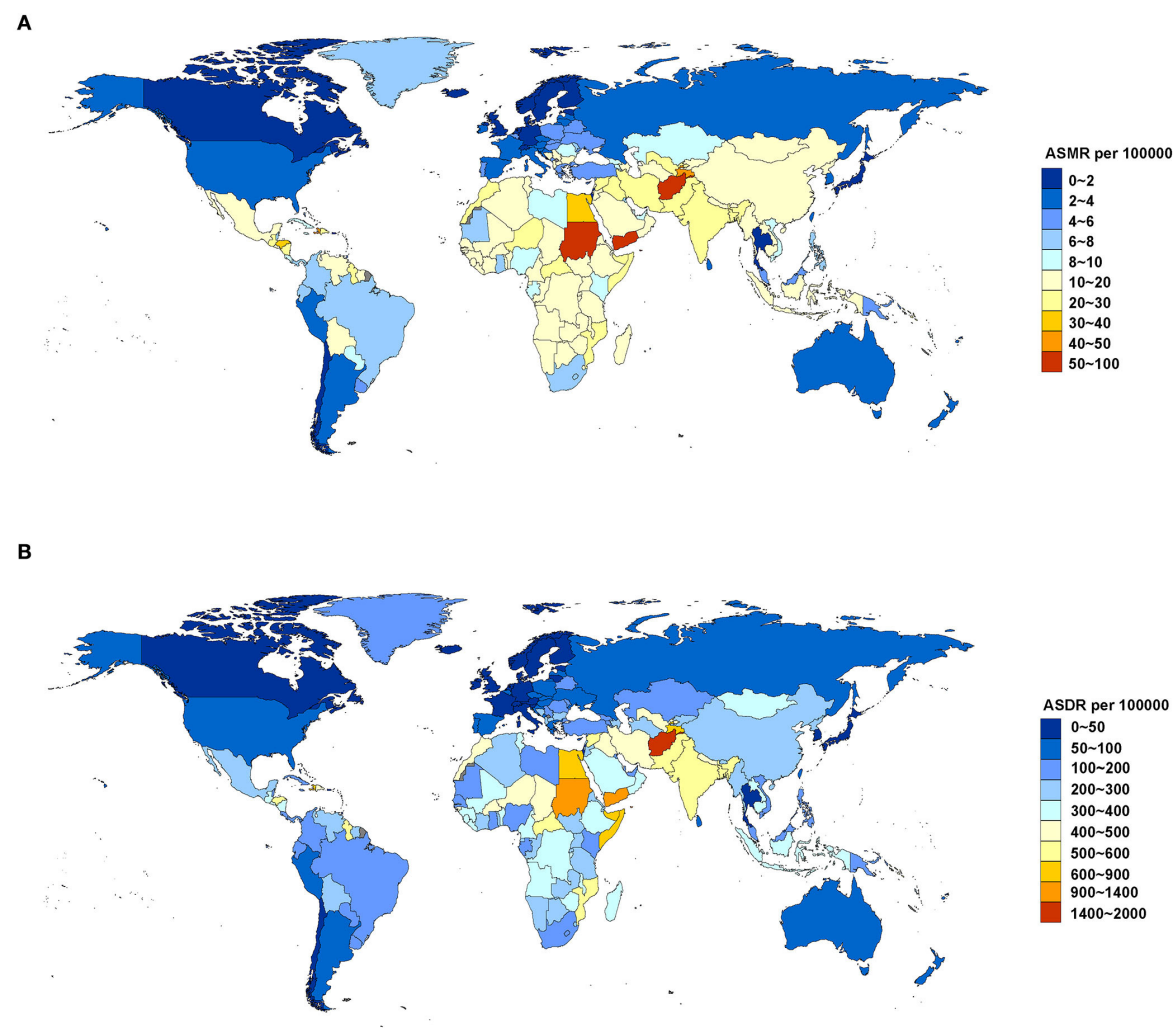
ASMR **(A)** and ASDR **(B)** attributable to lead exposure by country and territory in 2019. ASMR, age-standardized mortality rate; ASDR, age-standardized DALY rate.

For age-standardized rate, South Asia (23.75, 95% UI: 16.68, 31.48) and North Africa and the Middle East (23.36, 95% UI: 15.11, 33.28) were the regions with the highest ASMR (per 100,000 population) in 2019. The top five countries and territories of ASMR in 2019 were Afghanistan (82.791, 95% UI: 59.291, 113.164), Yemen (63.966, 95% UI: 44.328, 85.941), Sudan (50.275, 95% UI: 35.227, 70.316), Tajikistan (41.769, 95% UI: 25.544, 60.576), and Haiti (41.634, 95% UI: 27.421, 60.090) (Figure 2A; Supplementary Table 3). South Asia (576.94, 95% UI: 414.10, 751.70) and North Africa and Middle East (489.27, 95% UI: 320.52, 669.63) were also two GBD regions with the highest ASDR (per 100,000 population) in 2019. The top five countries and territories of ASDR were Afghanistan (1,869.909, 95% UI: 1,349.827, 2,485.584), Yemen (1,362.771, 95% UI: 971.755, 1,816.462), Sudan (1,041.621, 95% UI: 736.796, 1,816.462), Haiti (847.915, 95% UI: 543.243, 1,232.574), and Egypt (800.041, 95% UI: 500.904, 1,185.547) (Figure 2B; Supplementary Table 4).

**Figure 3:**
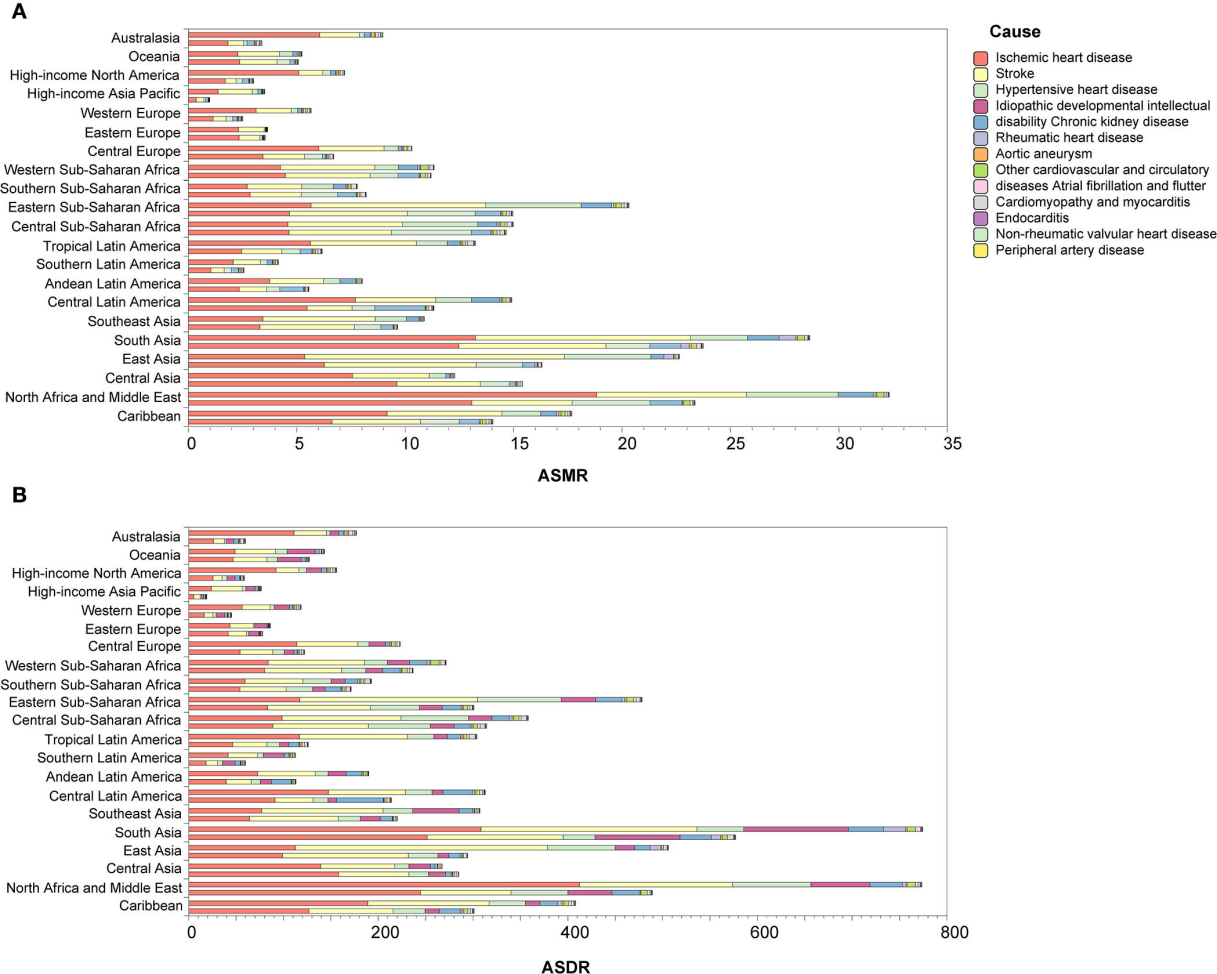
Changes of ASMR **(A)** and ASDR **(B)** in 1990 and 2019 by region. In each group, the upper column is for 1990 and the lower column is for 2019. ASMR, age-standardized mortality rate; ASDR, age-standardized disability-adjusted life year rate.

**Figure 4:**
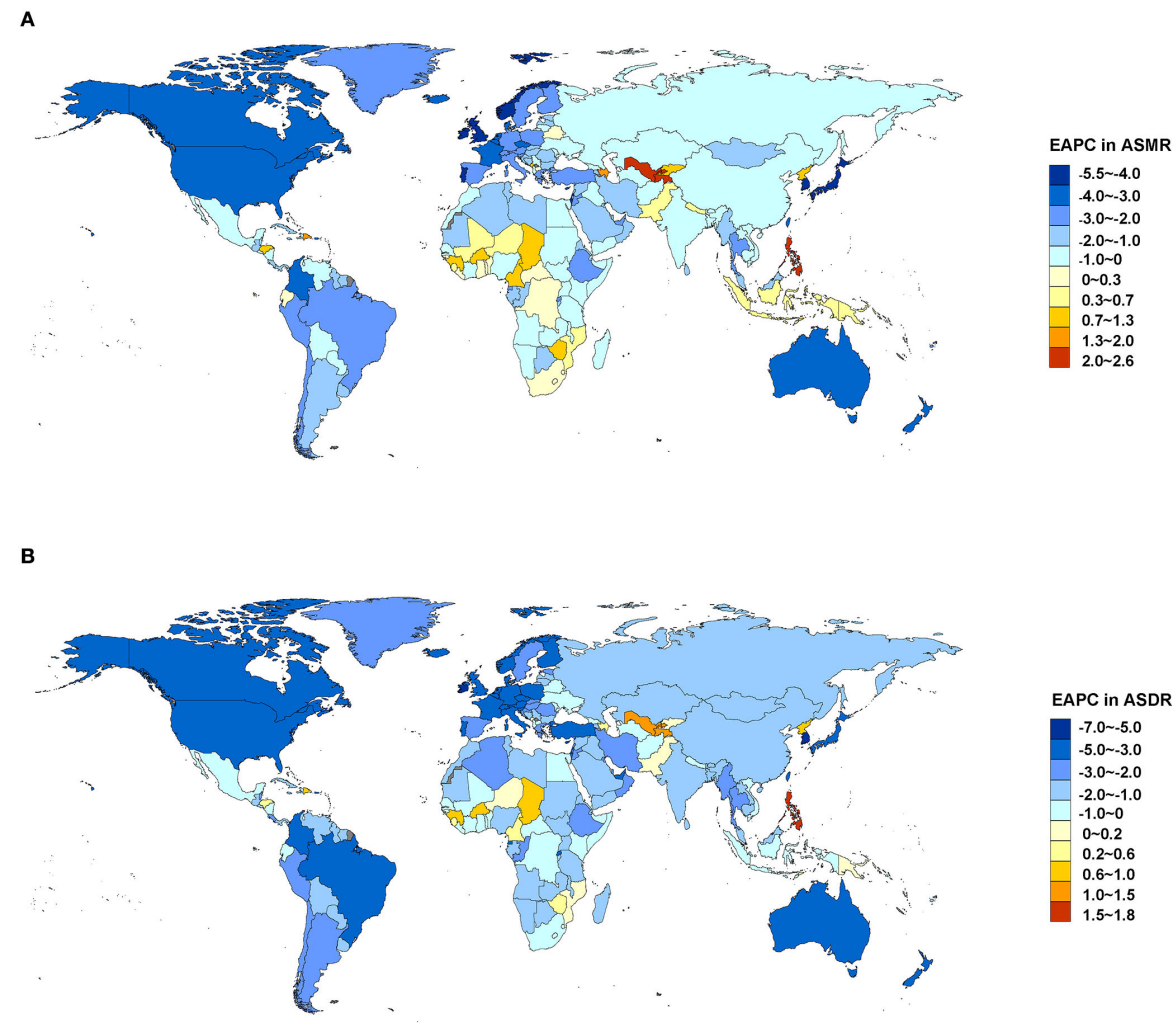
EAPC in ASMR **(A)** and ASDR **(B)** attributable to lead exposure from 1990 to 2019 by country and territory. EAPC, estimated annual percentage change; ASMR, age-standardized mortality rate; ASDR, age-standardized disability-adjusted life year rate.

Between 1990 and 2019, most regions of ASMR decreased except for Central Asia and Southern sub-Saharan Africa, and more than 50% declines were observed in high-income Asia Pacific (72.18%), Australasia (62.19%), high-income North America (58.22%), Western Europe (55.55%), and tropical Latin America (53.37%). For ASDR, only Central Asia increased, and more than 50% declines were observed in high-income Asia Pacific (75.06%), Australasia (66.16%), high-income North America (62.34%), Western Europe (61.71%), and tropical Latin America (58.47%) (Figure 3). At the country or territory level, a downward trend of ASMR and ASDR with values of EAPC <0 was identified in most countries and territories (Figure 4; Supplementary Tables 5, 6). The countries and territories with the largest drop of ASMR were the Republic of Korea, the United Kingdom, and Israel, and their EAPC were −5.46 (95% CI: −5.62, −5.30), −4.41 (95% CI: −4.62, −4.21), and −4.37 (95% CI: −4.52, −4.22), respectively. The Republic of Korea, Ireland, and Singapore showed the largest decline in ASDR with the EAPC of −6.32 (95% CI: −6.45, −6.19), −5.05 (95% CI: −5.26, −4.83), and −4.83 (95% CI: −4.98, −4.68), respectively. The highest increase of ASMR occurred in Uzbekistan, the Philippines, and Tajikistan with the EAPC of 2.52 (95% CI: 1.77, 3.28), 2.40 (95% CI: 1.88, 2.91), and 2.22 (95% CI: 1.90, 2.54), respectively. With some minor changes in rank order, the Philippines, Uzbekistan, and Tajikistan owned the highest increase of ASDR with the EAPC of 1.71 (95% CI: 1.20, 2.22), 1.40 (95% CI: 0.71, 2.11), and 1.22 (95% CI: 0.94, 1.49), respectively.

### Global burden of diseases attributable to lead exposure by sex and age

**Figure 5:**
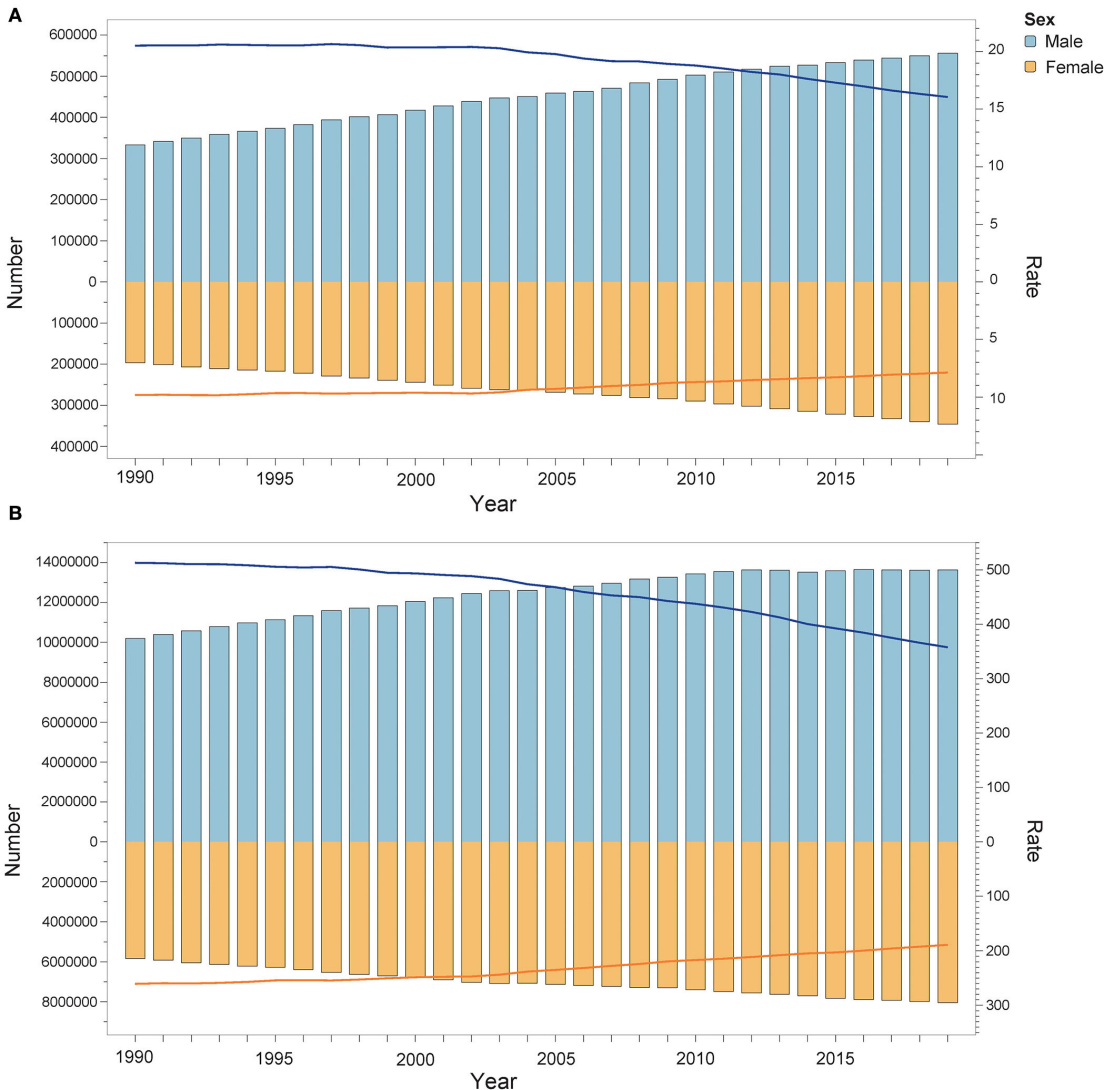
Deaths **(A)** and DALYs **(B)** attributable to lead exposure from 1990 to 2019 by sex. The bar shows the number of deaths and DALYs. The line indicates ASMR and ASDR. DALYs, disability-adjusted life years; ASMR, age-standardized mortality rate; ASDR, age-standardized DALY rate.

In 2019, the deaths attributable to lead exposure (10^3^) in men and women were 555.95 (95% UI: 357.23, 766.84) and 345.77 (95% UI: 188.45, 532.93), respectively. The DALYs (10^4^) in men and women were 1,363.37 (95% UI: 888.05, 1882.35) and 804.26 (95% UI: 481.80, 1172.77), respectively. For the age-standardized rate, the ASMR and ASDR (per 100,000 population) in men were also greater than in women. The ASMR attributable to lead exposure was 16.051 (95% UI: 10.255, 21.956) and 7.877 (95% UI: 4.289, 12.148) in men and women in 2019, respectively. The ASDR in men and women was 357.56 (95% UI: 233.38, 493.30) and 188.85 (95% UI: 113.07, 274.64) in 2019, respectively. Additionally, Figure 5 shows that from 1990 to 2019, the global disease burden attributable to lead exposure in men was always greater than in women. Although the number of deaths and DALYs were increasing, the ASMR and ASDR were both decreasing in both sexes, and a greater decline was observed in men. Specifically, between 1990 and 2019, the ASMR decreased by 21.77% and 19.86% in men and women, respectively, and the ASDR decreased by 30.28% and 27.51% in men and women, respectively (Supplementary Tables 7, 8).

**Figure 6:**
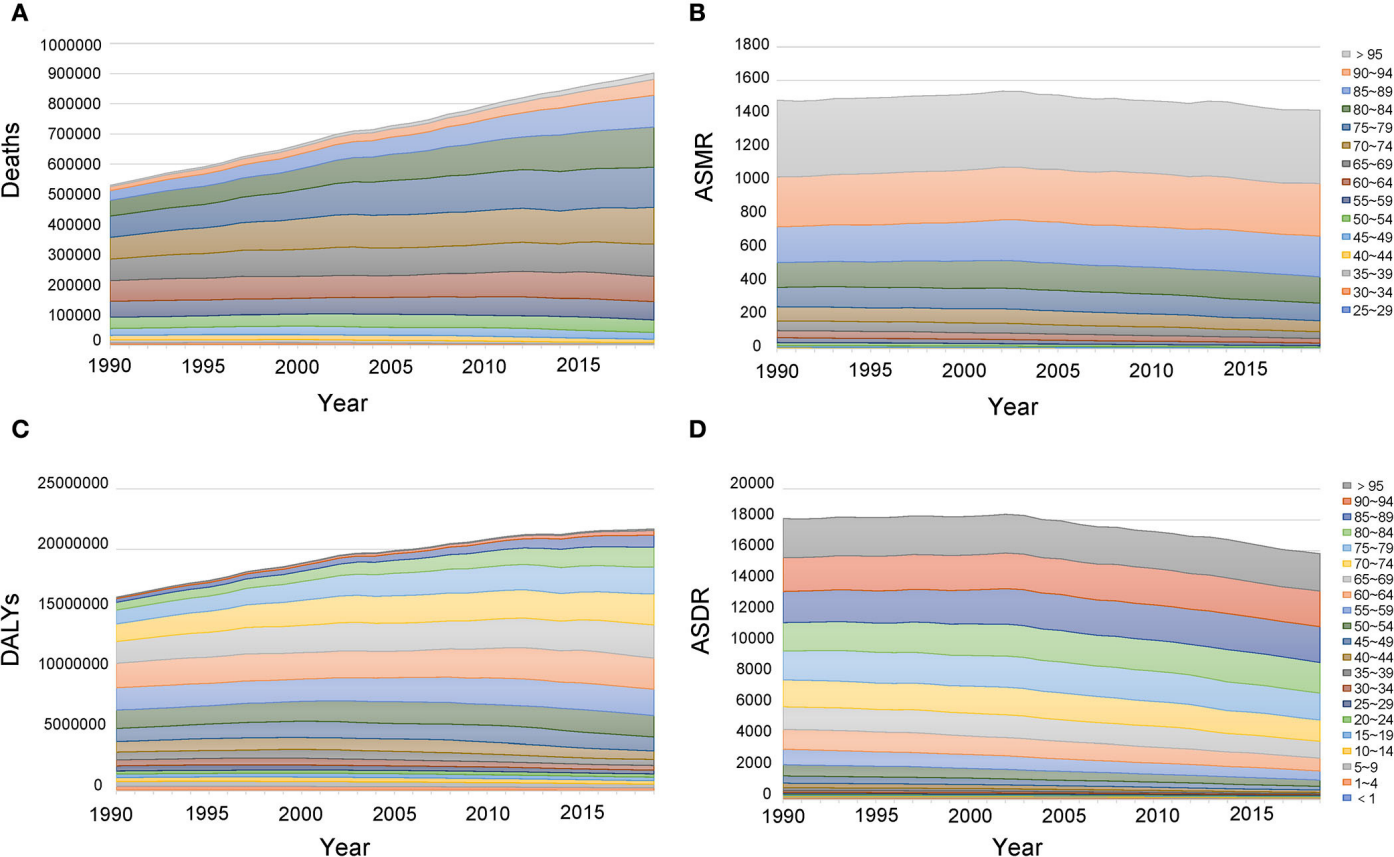
Age distribution of deaths **(A)**, ASMR **(B)**, DALYs **(C)**, and ASDR **(D)** attributable to lead exposure from 1990 to 2019. DALYs, disability-adjusted life years; ASMR, age-standardized mortality rate; ASDR, age-standardized DALY rate.

**Figure 7:**
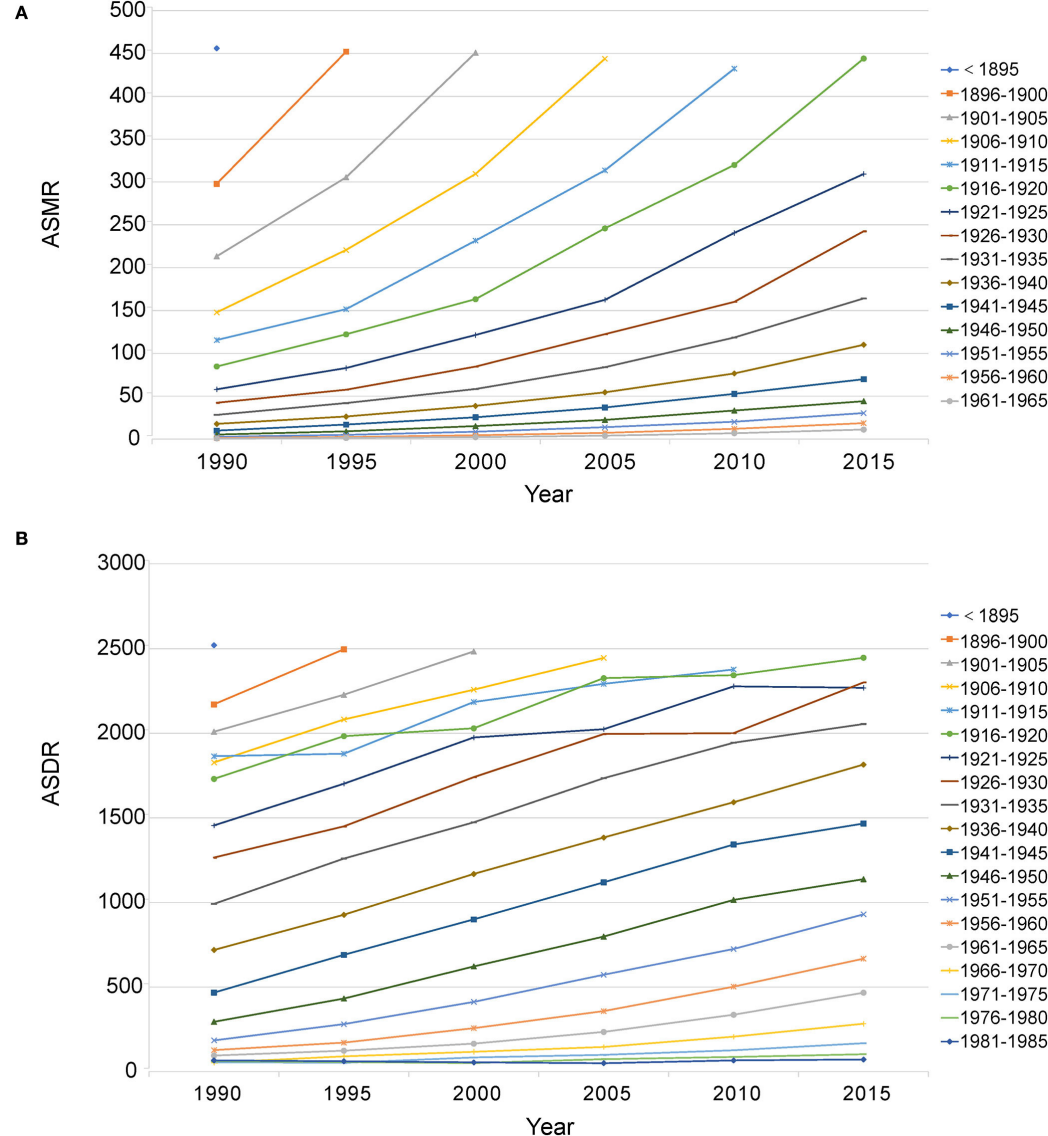
Birth cohort analysis of ASMR **(A)** and ASDR **(B)** attributable to lead exposure. ASMR, age-standardized mortality rate; ASDR, age-standardized DALY rate.

In 2019, most of the deaths occurred in the population aged from 60 to 89, accounting for 75.65% of total deaths. Most DALYs occurred in the population aged from 50 to 84, accounting for 72.50% of total DALYs. Between 1990 and 2019, the abovementioned age groups almost always had higher deaths and DALYs (Figure 6; Supplementary Tables 9, 10). For the age-standardized rate, in 2019, the ASMR and ASDR increased with age, and the highest age-standardized rates were both in age groups more than 95 years (ASMR: 436.26, 95%CI: 213.94, 719.42; ASDR: 2402.85, 95%CI: 1189.58, 3921.27). Birth cohort analysis showed that cohorts born earlier had higher ASMR and ASDR, and age-standardized rates increased with age in all birth cohorts (Figure 7).

### Association between SDI and disease burden attributable to lead exposure

**Figure 8:**
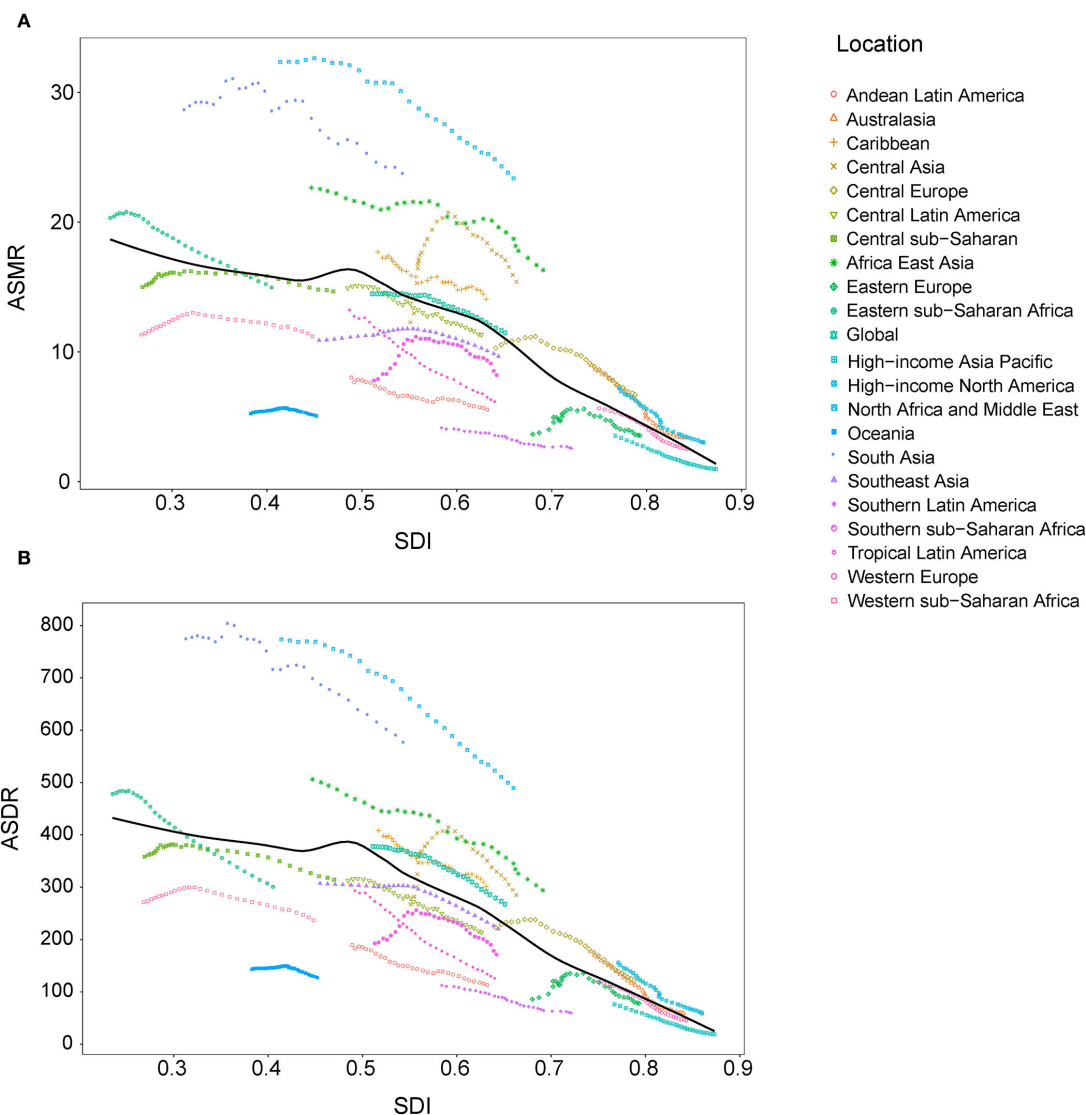
Correlations between SDI and ASMR **(A)** and ASDR **(B)** attributable to lead exposure from 1990 to 2019 by region. ASMR, age-standardized mortality rate; ASDR, age-standardized disability-adjusted life year rate; SDI, sociodemographic index.

**Figure 9:**
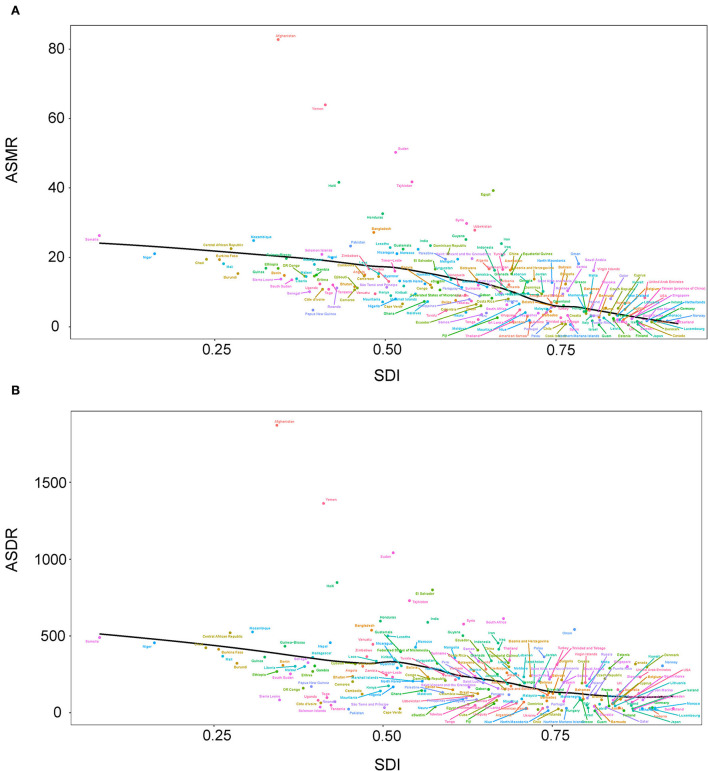
Correlations between SDI and ASMR **(A)** and ASDR **(B)** attributable to lead exposure in 2019 by country and territory. ASMR, age-standardized mortality rate; ASDR, age-standardized disability-adjusted life year rate; SDI, sociodemographic index.

**Figure 10:**
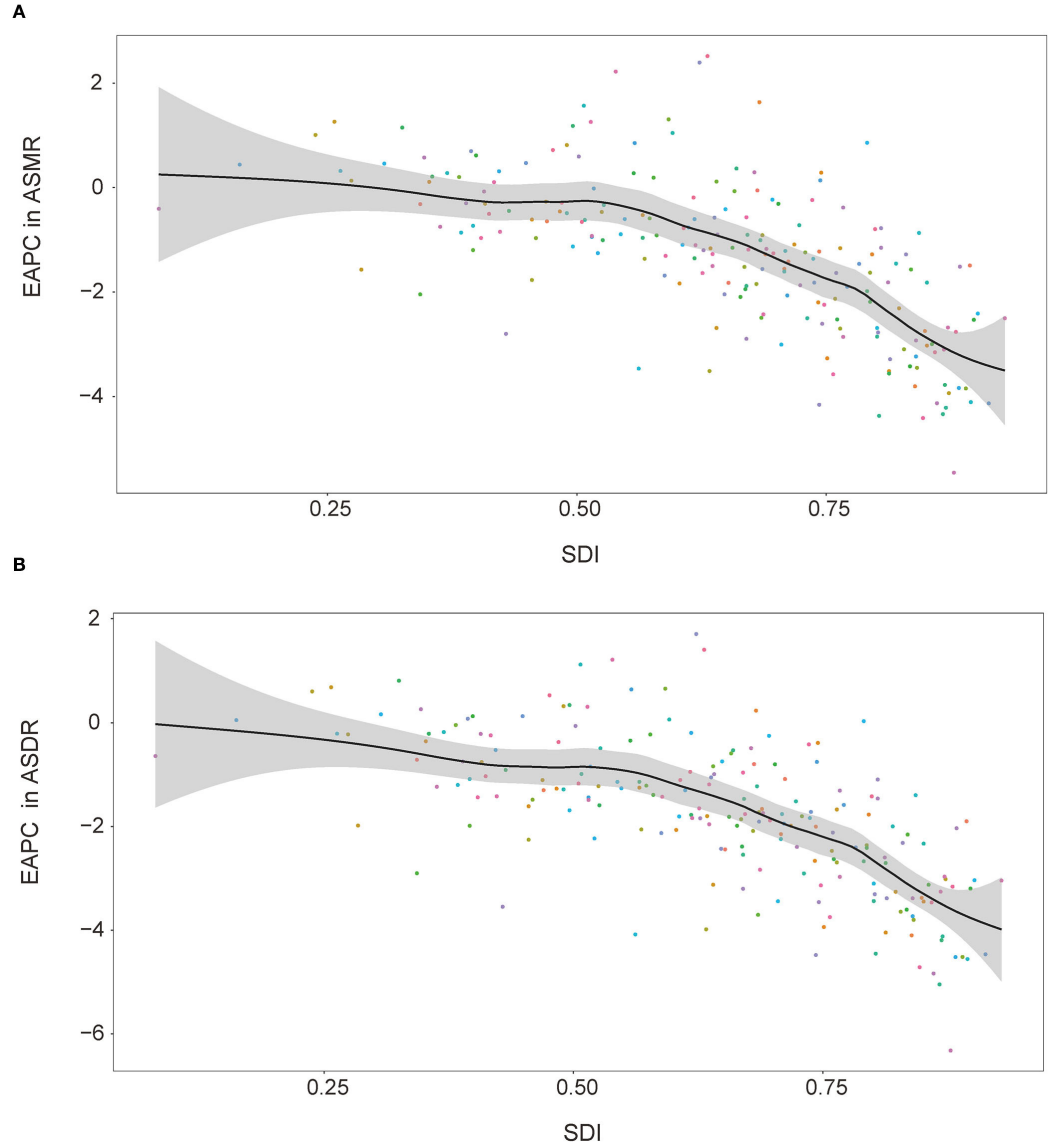
Correlations between SDI and EAPC in ASMR **(A)** and ASDR **(B)** attributable to lead exposure in 2019 by country and territory. EAPC, estimated annual percentage change; ASMR, age-standardized mortality rate; ASDR, age-standardized disability-adjusted life year rate; SDI, sociodemographic index.

At the regional level, the associations between SDI and age-standardized rates were similar. In most regions, the ASMR and ASDR showed a negative correlation and decreased with the SDI value. But there was an inverse-V shape relation in Central Asia, southern sub-Saharan Africa, and Eastern Europe (Figure 8). In 2019, an inverse association between age-standardized rates and SDI at the country and territory levels was observed (Figure 9). Similar patterns were recognized for most causes of GBD Level 3, except for peripheral artery disease and non-rheumatic valvular heart disease (Supplementary Figures 1, 2). The EAPC in ASMR and ASDR presented a significant negative correlation with the SDI in 2019, and the correlation coefficients were −0.699 and −0.697, respectively (Figure 10).

## Discussion

Lead can cause harmful systemic effects on human health and remains a risk factor for many diseases. In this study, we evaluated the temporal-spatial distribution of disease burden attributable to lead exposure by analyzing data from GBD 2019. From 1990 to 2019, the total deaths and DALYs both showed a considerable increase because of population growth and aging. It is a high probability that the absolute number will continue to grow, indicating that lead exposure will be a long-term threat to human health for a period of time in the future. For GBD Level 2 cause, the majority of deaths and DALYs of lead exposure were from cardiovascular diseases. Higher lead exposure was associated with hypertension in the general population (22), and high systolic blood pressure is the most significant mediated risk factor for cardiovascular diseases (23). These contributed to the higher proportion of cardiovascular diseases in the disease burden of lead exposure. Some epidemiological studies based on the National Health and Nutrition Examination Survey (NHANES) have also revealed the adverse effects of lead exposure on the cardiovascular system (8, 24–28). For example, an analysis of NHANES III data showed that the increased blood lead level was associated with cardiovascular disease mortality (HR: 1.70; 95% CI: 1.30–2.22) (29). Our results from these studies highlight the health hazards of lead exposure on the cardiovascular system. For GBD Level 3 cause, the deaths and DALYs of chronic kidney disease were greater than most cardiovascular diseases, except for ischemic heart disease, stroke, and hypertensive heart disease. Lead exposure and high systolic blood pressure are both individual risk factors for chronic kidney disease (30), thus higher disease burden of chronic kidney disease is not surprising.

For age-standardized rates, the overall ASMR and ASDR declined in the past decades. This may be partially ascribed to the decreasing lead exposure level caused by the improvement in awareness of lead poisoning and the limited use of leaded products. The geometric mean blood lead level decreased from 12.8 to 0.82 μg/dl in the population of the United States aged 1–74 years between 1976–1980 and 2015–2016 (31). GBD 2019 Risk Factors Collaborators estimated that the summary exposure values (SEVs) of lead exposure declined significantly, and the annualized rate of change was −1.00% (−1.43 to −0.63) from 1990 to 2019 (18). Nevertheless, increasing age-standardized rates were observed in atrial fibrillation and flutter and chronic kidney disease. This phenomenon should be given more attention. Low-level environmental lead was reported to be associated with accelerated deterioration of chronic renal insufficiency, even at levels far below the limits of normal ranges (32). We speculate that the mechanisms of lead-associated diseases vary, and the thresholds for these are diverse. The lower lead damage threshold of atrial fibrillation and flutter and chronic kidney disease may induce the increase of ASMR and ASDR of them although lead exposure had dropped drastically.

The spatial variations were viewed as an epidemiological feature of the distribution of disease burden associated with lead exposure. In the regions or countries and territories with a large population, the deaths and DALYs are relatively higher. More importantly, age-standardized rates still increase in some Asian and African countries, and there are few published studies about lead exposure in these countries and territories. This also reminds us that research on lead exposure and health effects should be performed to explore the possible reasons for the increasing age-standardized rates in these countries and territories. In terms of sex, the effect of lead exposure on human health is sex-dependent (33). Genetic, physiological, and behavioral differences may contribute to it. In this study, the disease burden of lead exposure presented homogeneity by sex, and the absolute number and age-standardized rate in men were all greater than in women. The results of age distribution showed that the elderly population had a higher disease burden. These results indicate that older male individuals should be paid more attention when taking measures of prevention and treatment of lead exposure. The increasing age-standardized rates with age growing by birth cohort analysis also reveal the accumulating effects of lead exposure on human health. When comparing different birth cohorts, cohorts born earlier presented higher ASMR and ASDR. The lower exposure levels in the population born later due to the control measure of lead exposure at the end of the last century may contribute to the lower age-standardized rates in the later cohorts.

The negative associations between SDI and age-standardized rates were observed at the regional and country or territory levels. SDI is a proxy for multiple variables, including industrialization and healthcare level. In the countries with higher SDI, prevention and control measures, personal safety awareness, and the safety quality of food and water are better than in the countries with lower SDI. Importantly, these introduce a new issue—inequitable lead exposure (34–36). The average blood lead level, especially in children, seems to be higher in low-income and middle-income countries than in high-income countries (37). The largest economic costs of childhood lead exposure also happened in low- and middle-income countries (38). These environmental and social injustices were not only observed at the regional level like the abovementioned negative association between disease burden and SDI in this study but also within the country. For example, approximately 60% of children with confirmed elevated blood lead levels were non-Hispanic black in the United States from 1997 to 2001 (39). Geographic concentrations and social stratification may be used as a theoretical basis to guide the control and management of lead exposure.

There are some limitations to this study. First, the lead exposure level was estimated based on published data from 84 countries (18), and data from high-income countries may be given more weight. Second, although TMREL of lead exposure in GBD 2019 is lower than the reference value of 5 μg/dl established in 2012 by the Centers for Disease Control and Prevention of the United States and updated to 3.5 μg/dl using new NHANES data in 2021 (40), there is no known safe threshold of blood lead level, and the disease burden of lead exposure may be underestimated. TMREL ought to be lowered, and this change should be evaluated for the GBD 2020 cycle. Third, besides the cardiovascular, renal, and nervous systems, lead exposure can also damage other systems. Diseases concerned in GBD 2019 may be only the “tip of the iceberg”, and the disease burden attributable to lead exposure in the real world may be underestimated.

## Conclusion

Lead has been widely used all over the world since ancient times. Although age-standardized mortality and DALY rates attributable to lead exposure showed decrements in the past decades, the total deaths and DALYs were increasing and mainly occurred in the male and elderly population. SDI-associated environmental injustice of lead exposure induced the spatial variations of disease burden. Lead exposure is still a significant threat to public health on a global scale. Prevention and control measures should be taken in both developing and developed countries. The effect of secondary prevention on lead toxicity is limited. Primary prevention measures of identifying sources of lead and eliminating this toxin in the environment are more important, and these need interdisciplinary and interdepartmental cooperation.

## Data availability statement

Data sources are available in Global Health Data Results Tool (GHDx, http://ghdx.healthdata.org/gbd-results-tool). The original contributions presented in the study are included in the article/Supplementary material, further inquiries can be directed to the corresponding authors.

## Author contributions

NZ: conceptualization, data curation, funding acquisition, methodology, software, and writing—original draft. YH: methodology and visualization. ML: data curation, methodology, and software. LZ: formal analysis, supervision, and writing—reviewing and editing. HJ: conceptualization and writing–reviewing and editing. All authors contributed to the article and approved the submitted version.

## Funding

This study was supported by the National Natural Science Foundation of China (82003513) and the Zhishan Scholar Program of Southeast University (2242022R40062).

## Conflict of interest

The authors declare that the research was conducted in the absence of any commercial or financial relationships that could be construed as a potential conflict of interest.

## Publisher's note

All claims expressed in this article are solely those of the authors and do not necessarily represent those of their affiliated organizations, or those of the publisher, the editors and the reviewers. Any product that may be evaluated in this article, or claim that may be made by its manufacturer, is not guaranteed or endorsed by the publisher.
