# Supplementary material for: Trends in global burden of diseases attributable to lead exposure in 204 countries and territories from 1990 to 2019
Source: Front Public Health. 2022 Nov 23;10:1036398. doi: 10.3389/fpubh.2022.1036398 (PMC9727290; doi:10.3389/fpubh.2022.1036398)
Supplement: Supplementary file 2 [file Data_Sheet_2.pdf]

Figure 1 Association between age-standardized mortality rate (y axis) and SDI (x axis) at the country and territory level in 2019 by GBD Level 3 cause. SDI: sociodemographic

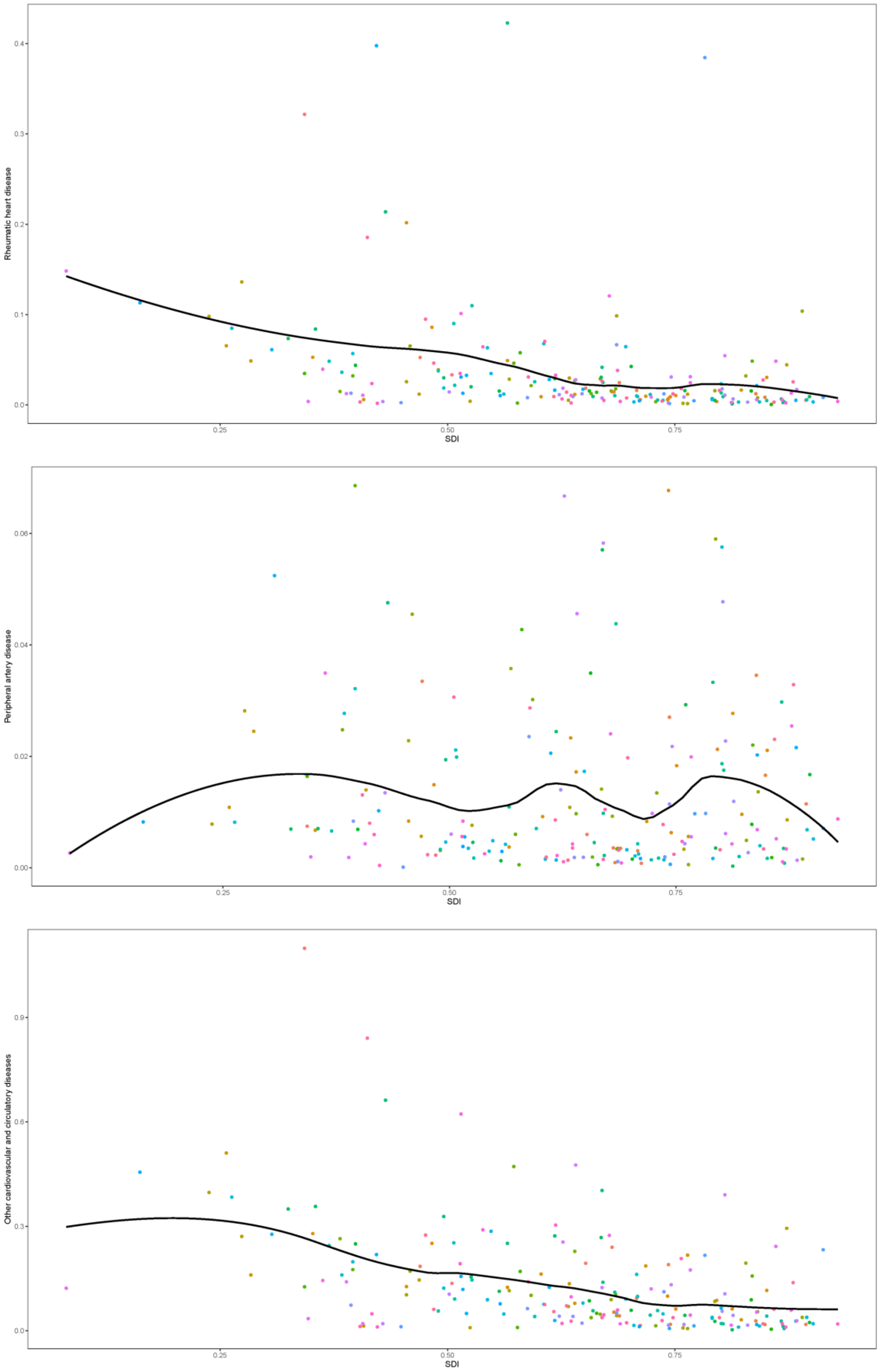

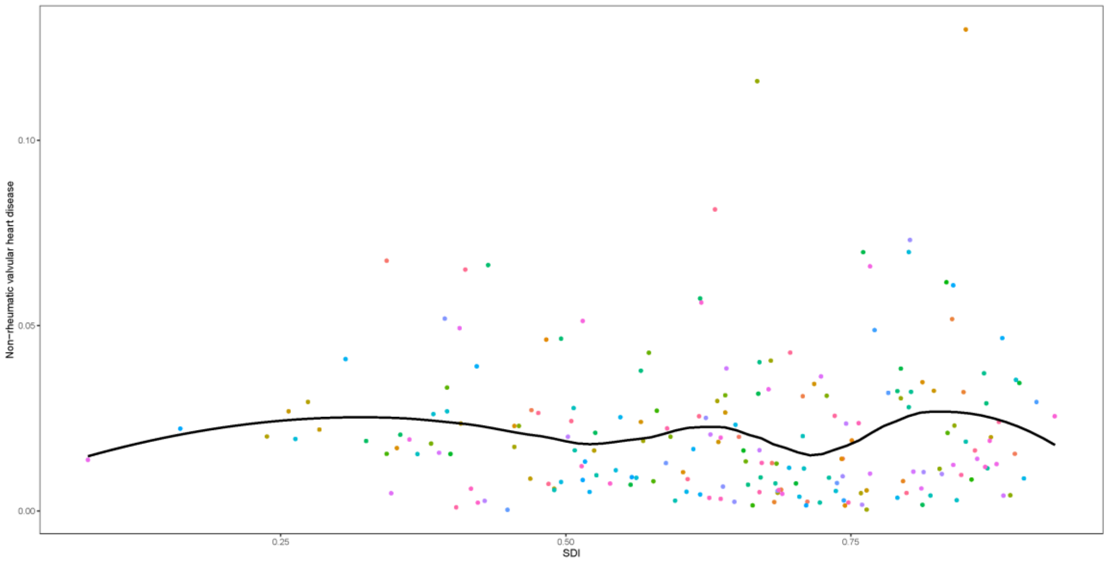

0.10
0.05
0.00
Non−rheumatic valvular heart disease
0.25
0.50
0.75
SDI

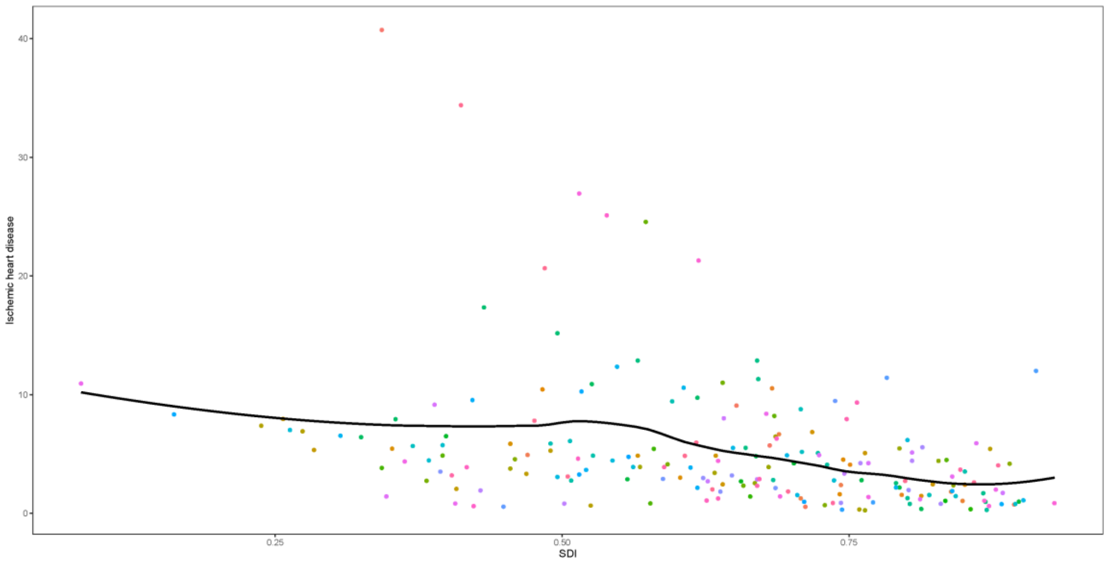

40
30
20
10
0
Ischemic heart disease
0.25
0.50
0.75
SDI

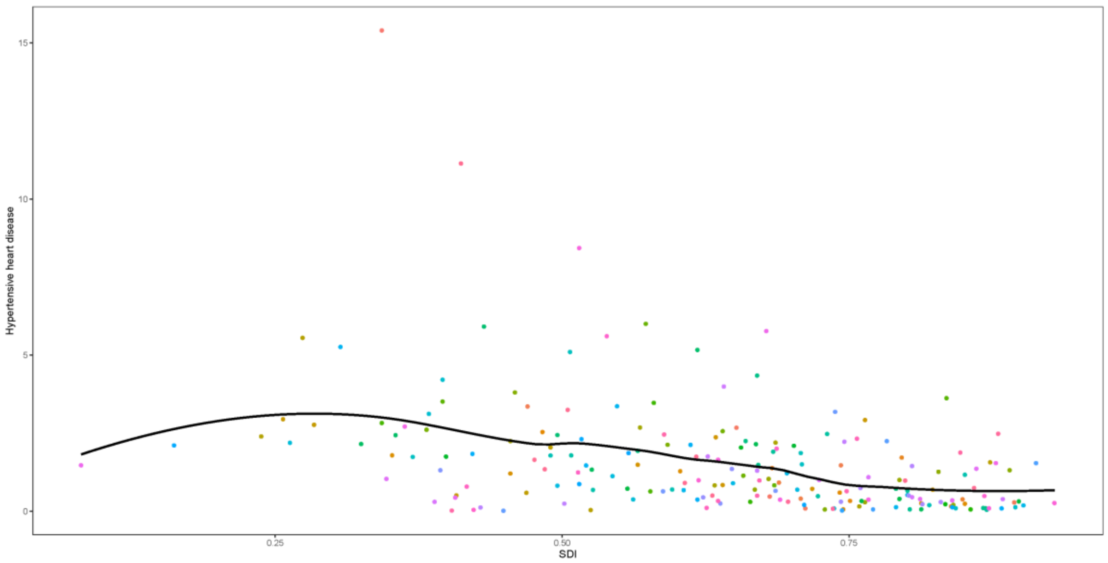

15
10
5
0
Hypertensive heart disease
0.25
0.50
0.75
SDI

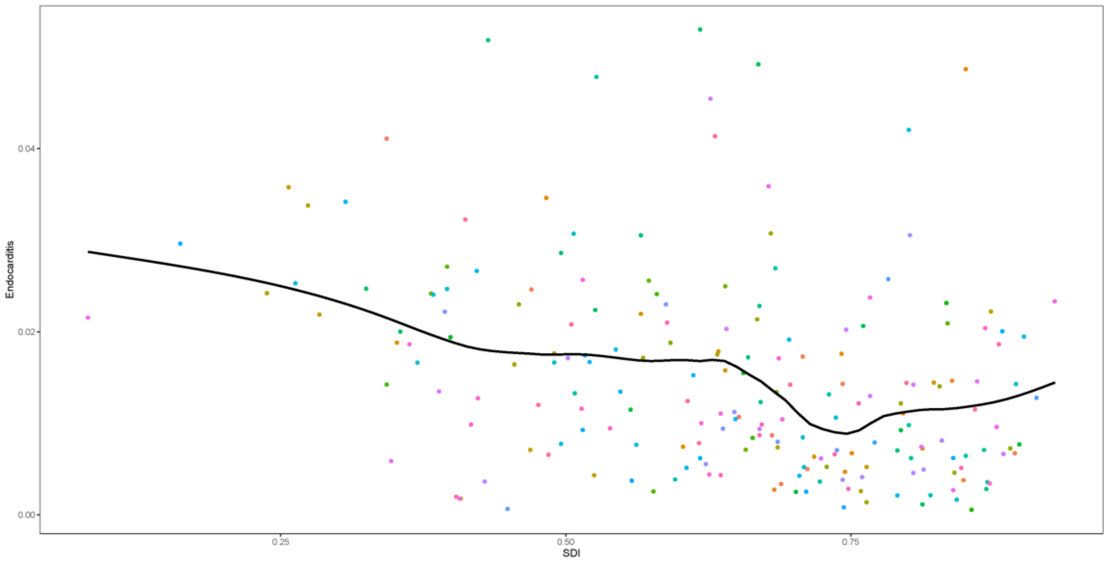
Endocarditis
0.00
0.02
0.04
0.25
0.50
0.75
SDI

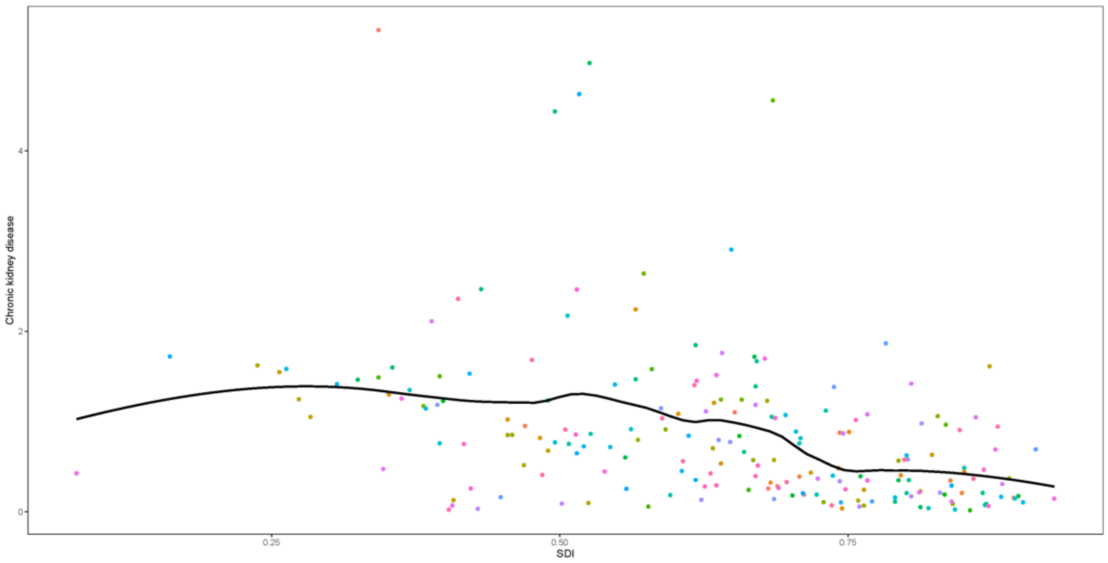
Chronic kidney disease
0
2
4
0.25
0.50
0.75
SDI

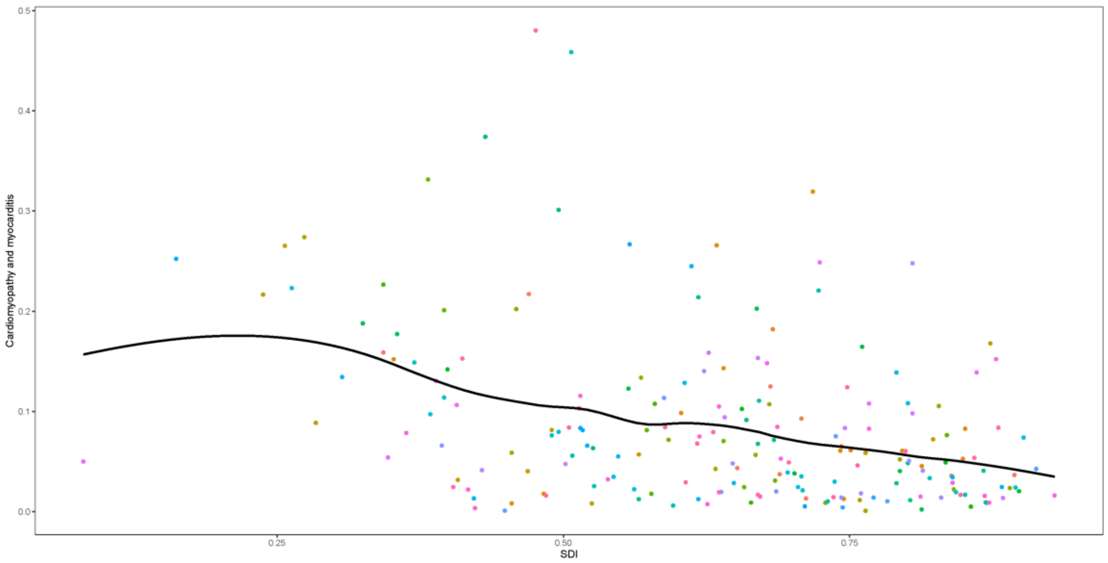
Cardiomyopathy and myocarditis
0.0
0.1
0.2
0.3
0.4
0.5
0.25
0.50
0.75
SDI

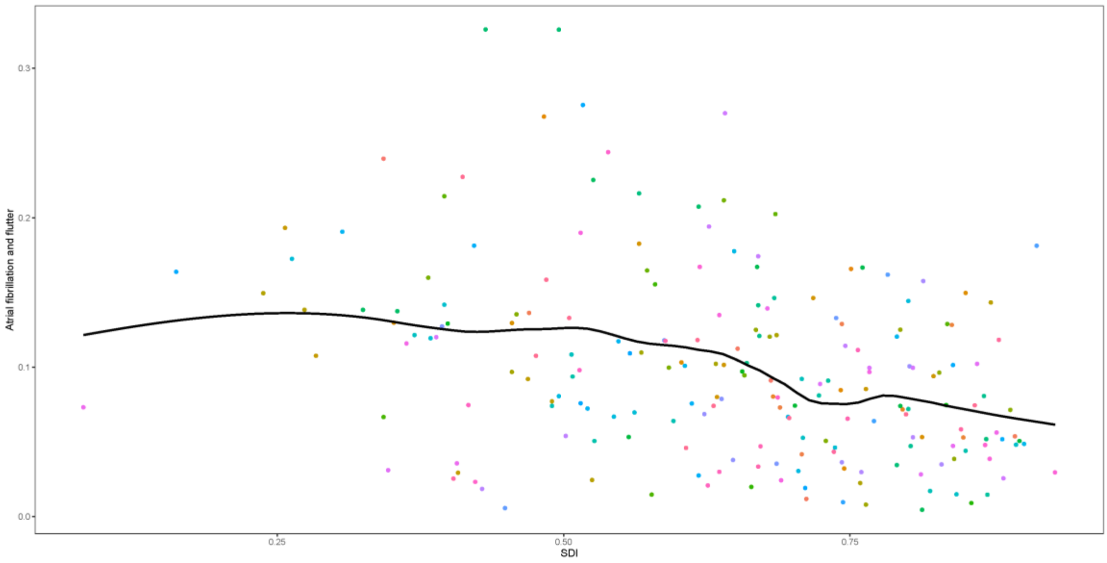
Atrial fibrillation and flutter
0.0
0.1
0.2
0.3
0.25
0.50
0.75
SDI

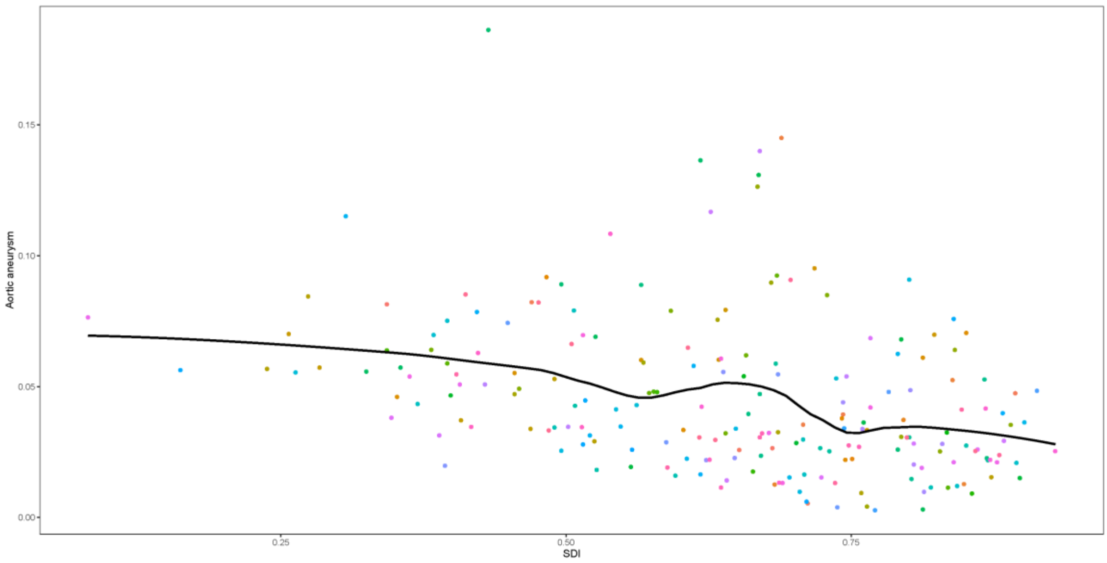
Aortic aneurysm
0.00
0.05
0.10
0.15
0.25
0.50
0.75
SDI

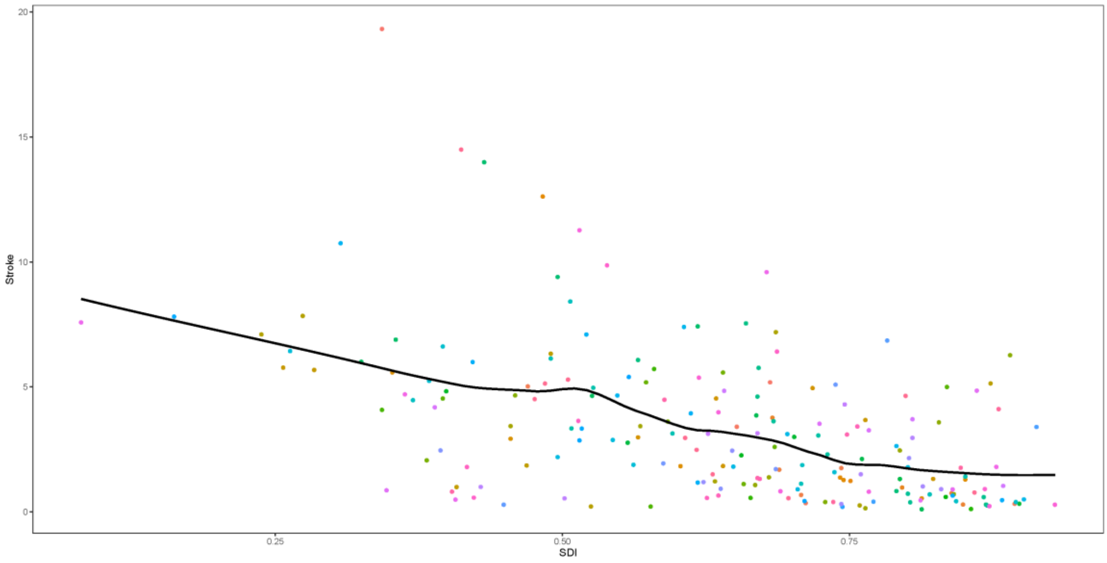
Stroke
0
5
10
15
20
0.25
0.50
0.75
SDI

Figure 2 Association between age-standardized disability-adjusted life year rate (y axis) and SDI (x axis) at the country and territory level in 2019 by GBD Level 3 cause. SDI: sociodemographic index.

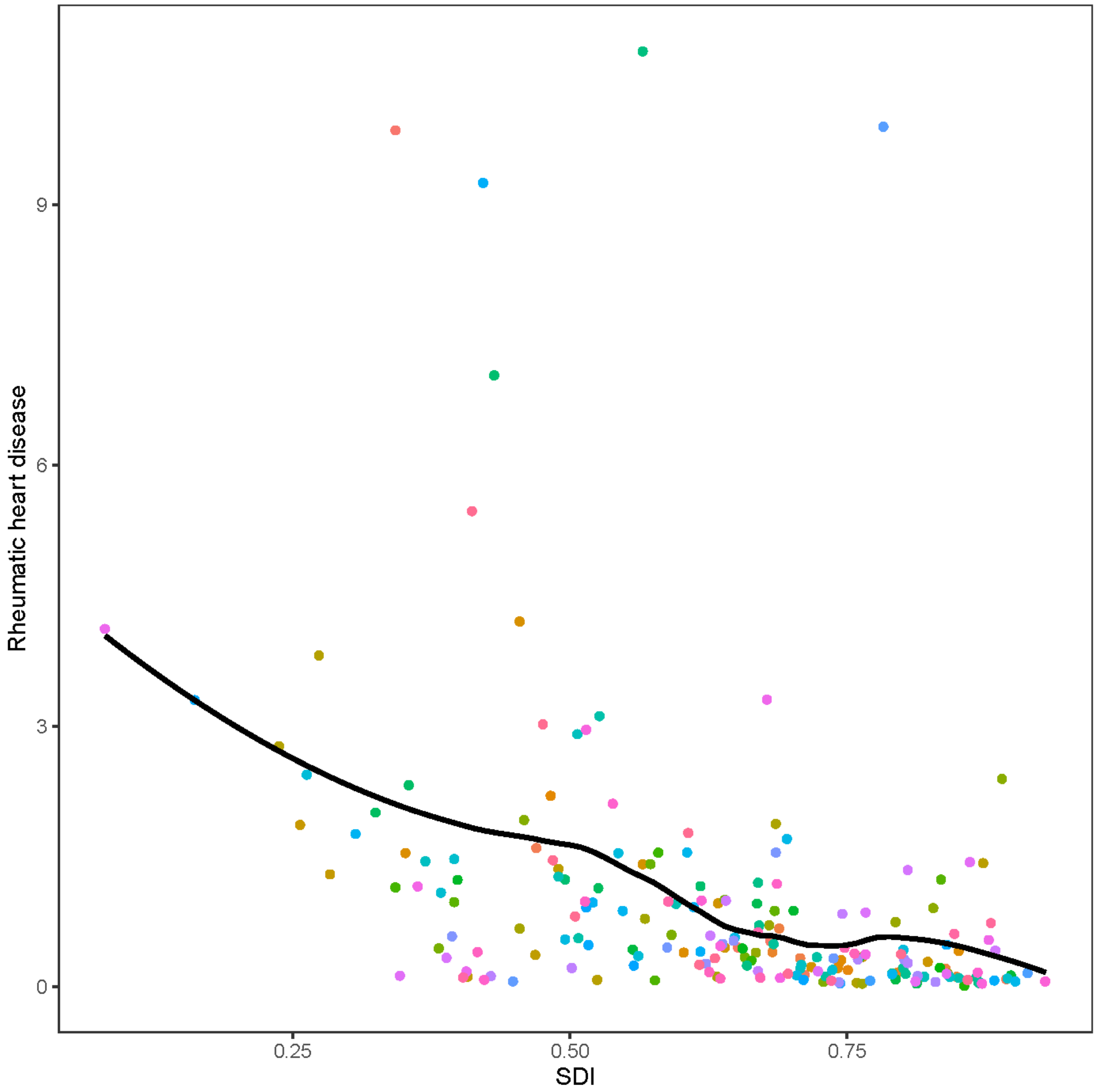

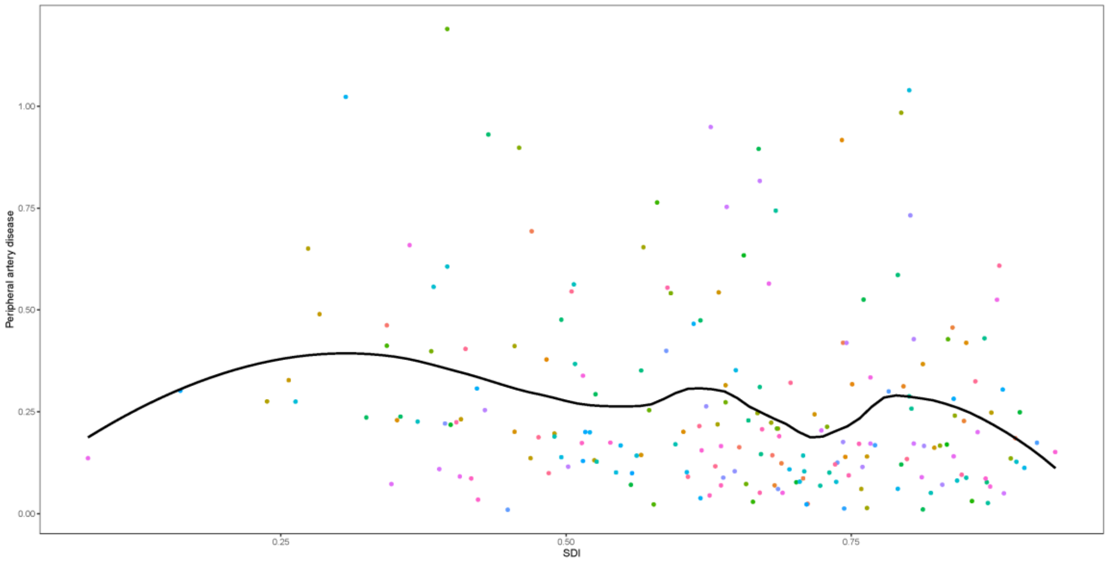

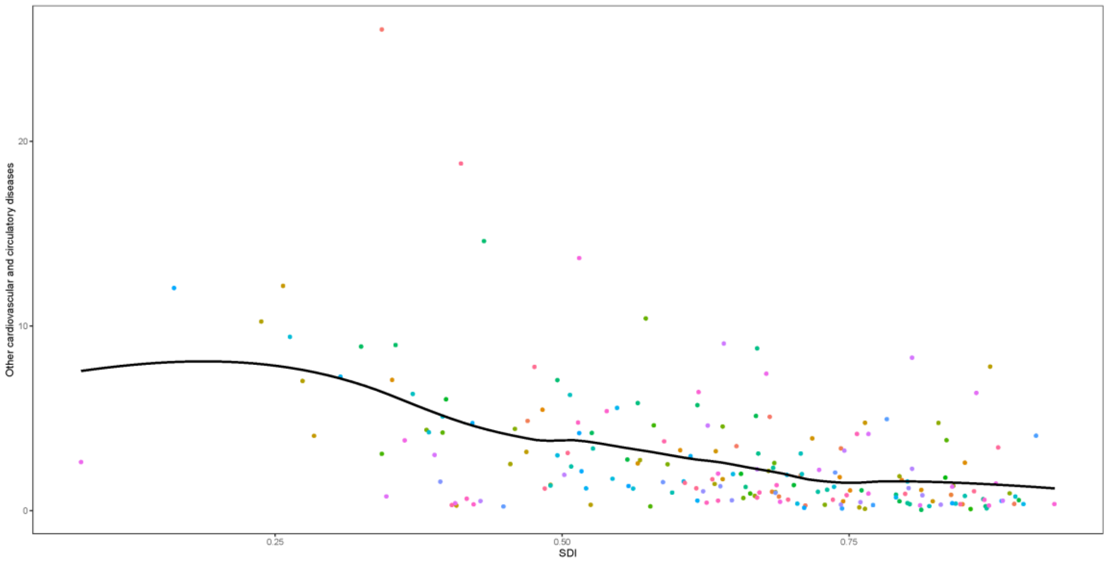
Other cardiovascular and circulatory diseases
20
10
0
0.25
0.50
0.75
SDI

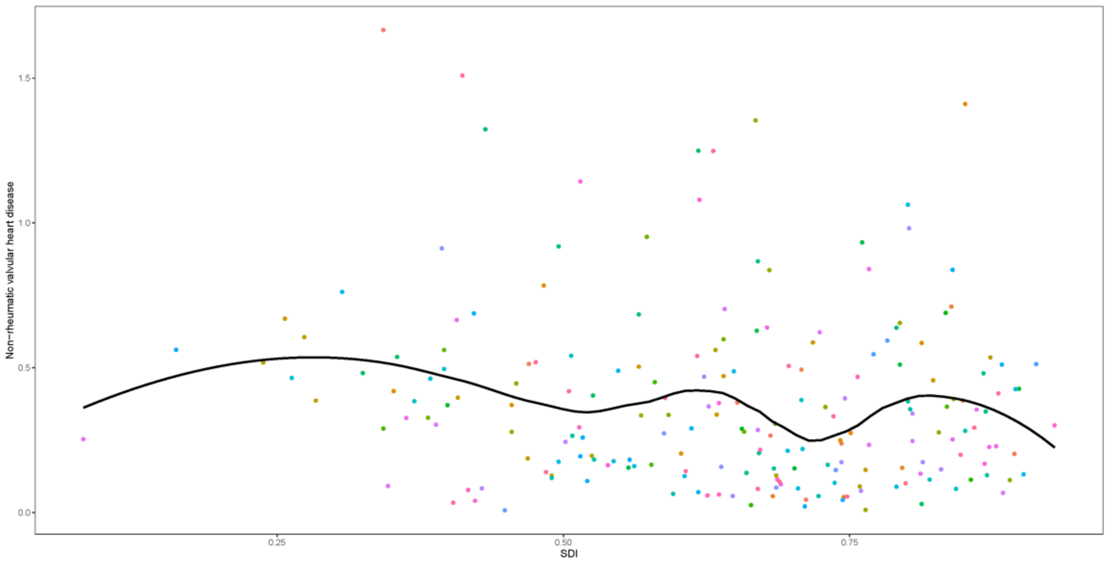
Non−rheumatic valvular heart disease
1.5
1.0
0.5
0.0
0.25
0.50
0.75
SDI

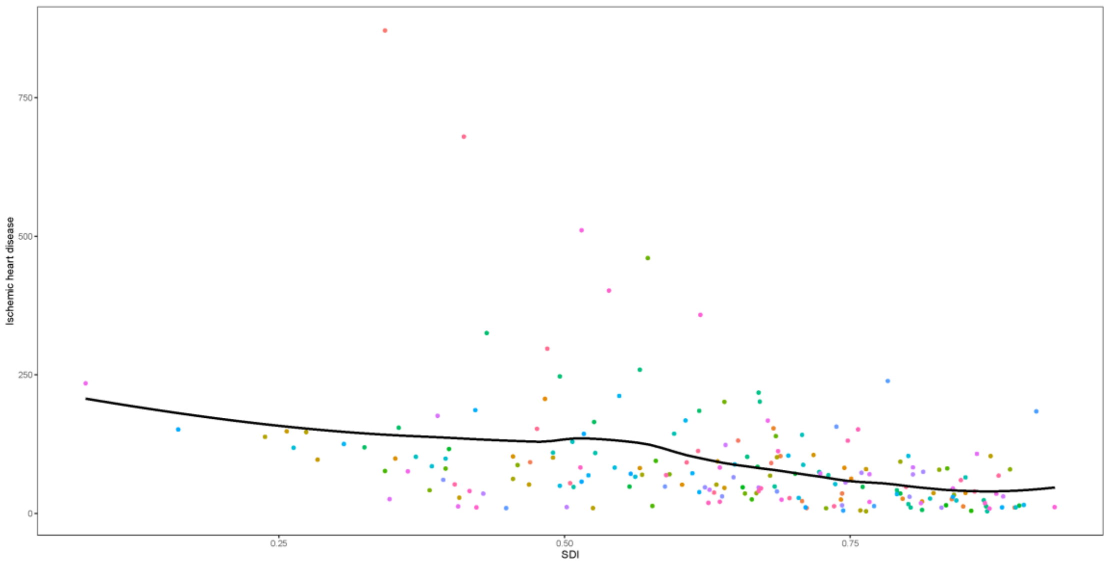
Ischemic heart disease
750
500
250
0
0.25
0.50
0.75
SDI

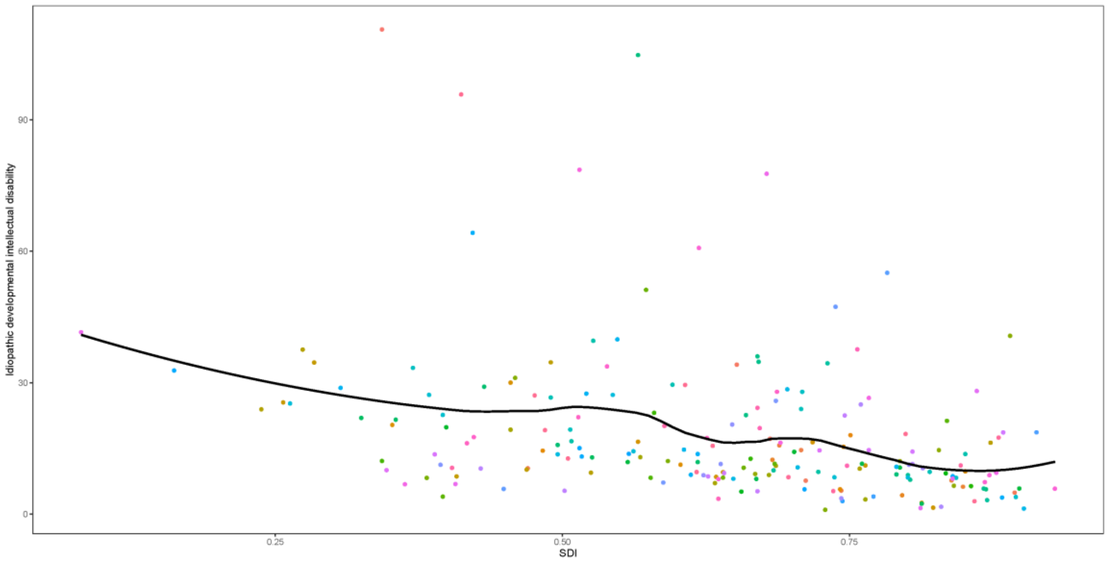
Idiopathic developmental intellectual disability
0
30
60
90
0.25
0.50
0.75
SDI

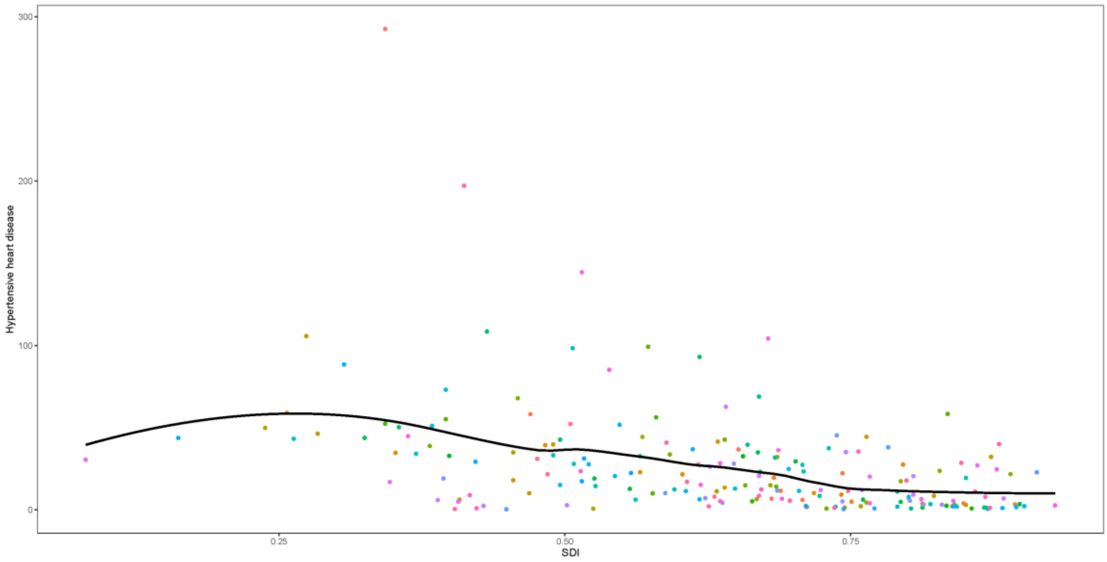
Hypertensive heart disease
0
100
200
300
0.25
0.50
0.75
SDI

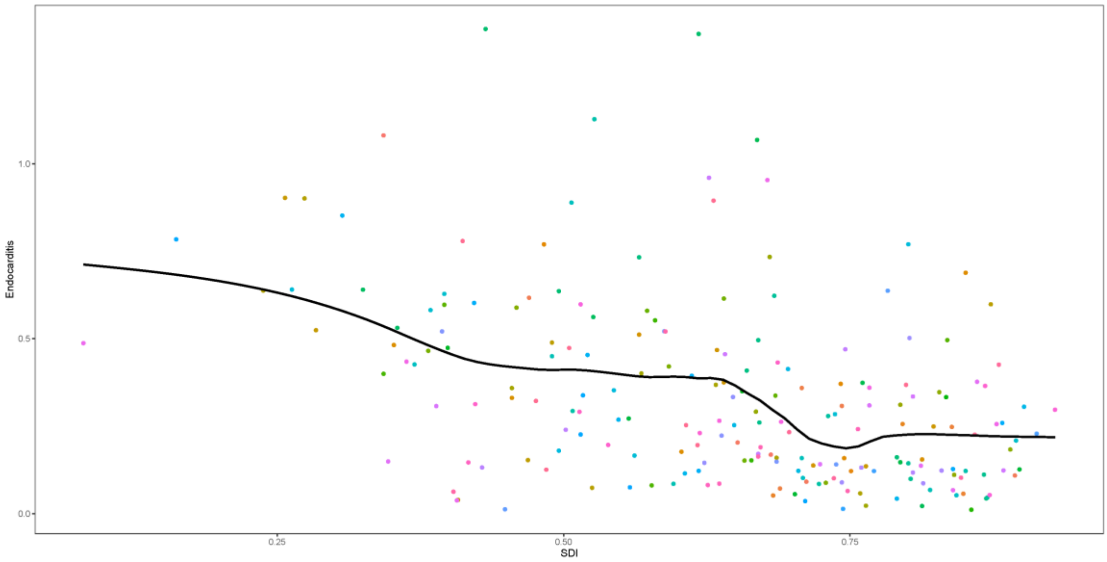
Endocarditis
0.0
0.5
1.0
0.25
0.50
0.75
SDI

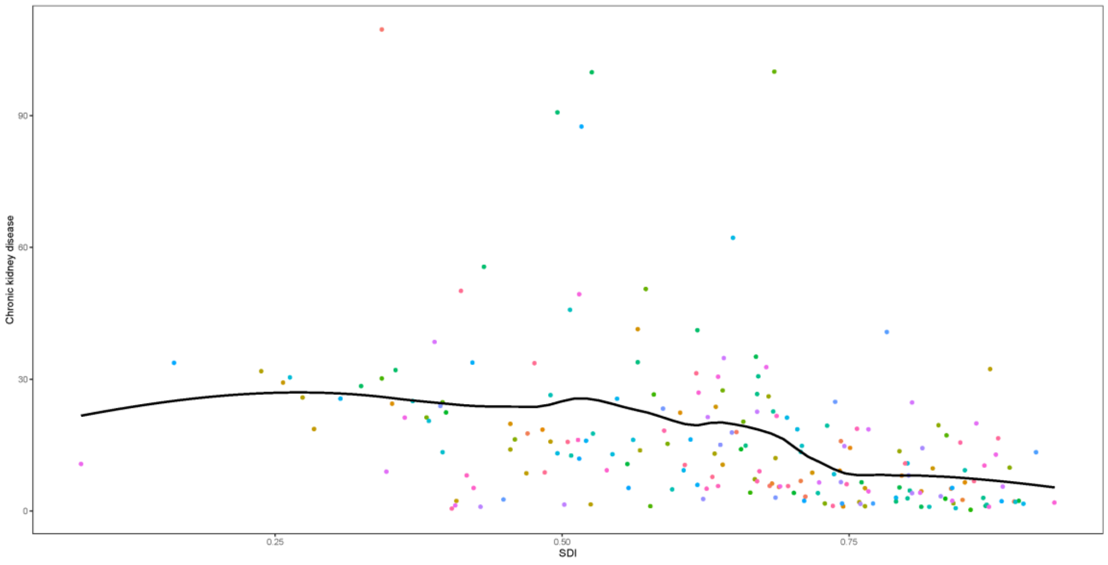
Chronic kidney disease
90
60
30
0
0.25
0.50
0.75
SDI

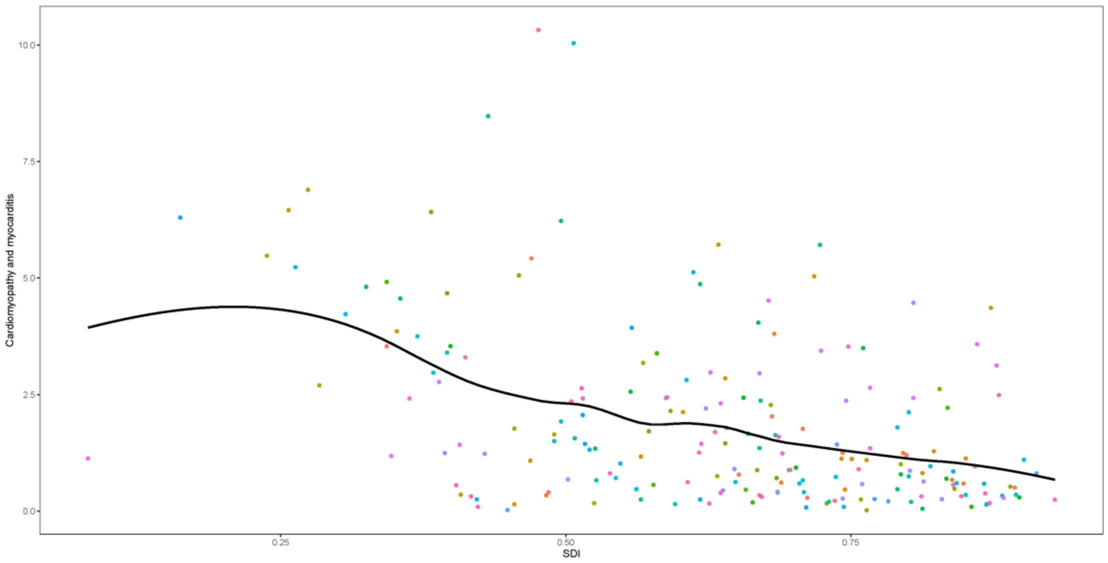
Cardiomyopathy and myocarditis
10.0
7.5
5.0
2.5
0.0
0.25
0.50
0.75
SDI

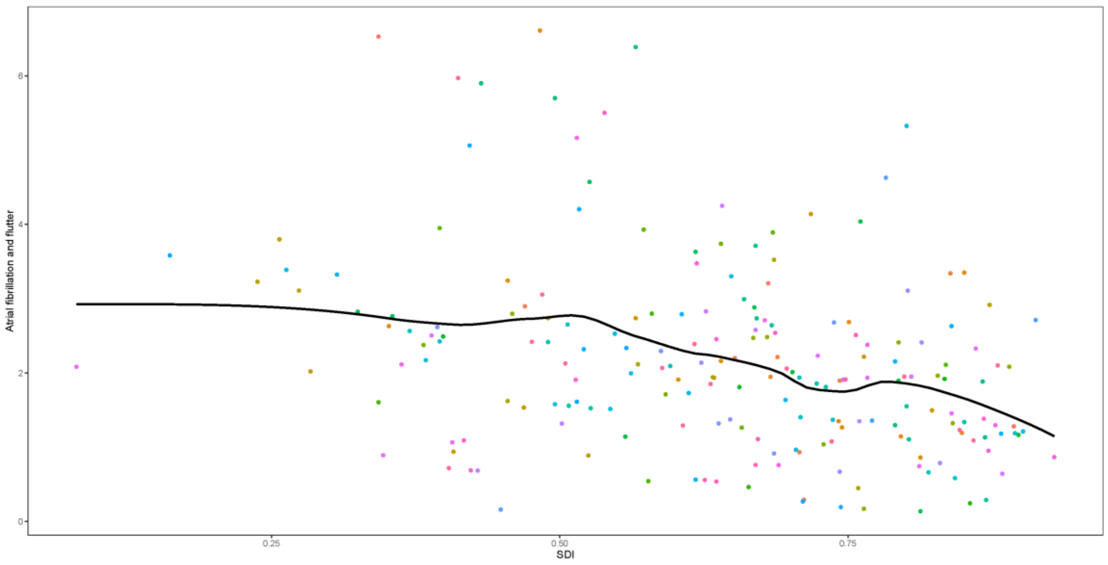
Atrial fibrillation and flutter
6
4
2
0
0.25
0.50
0.75
SDI

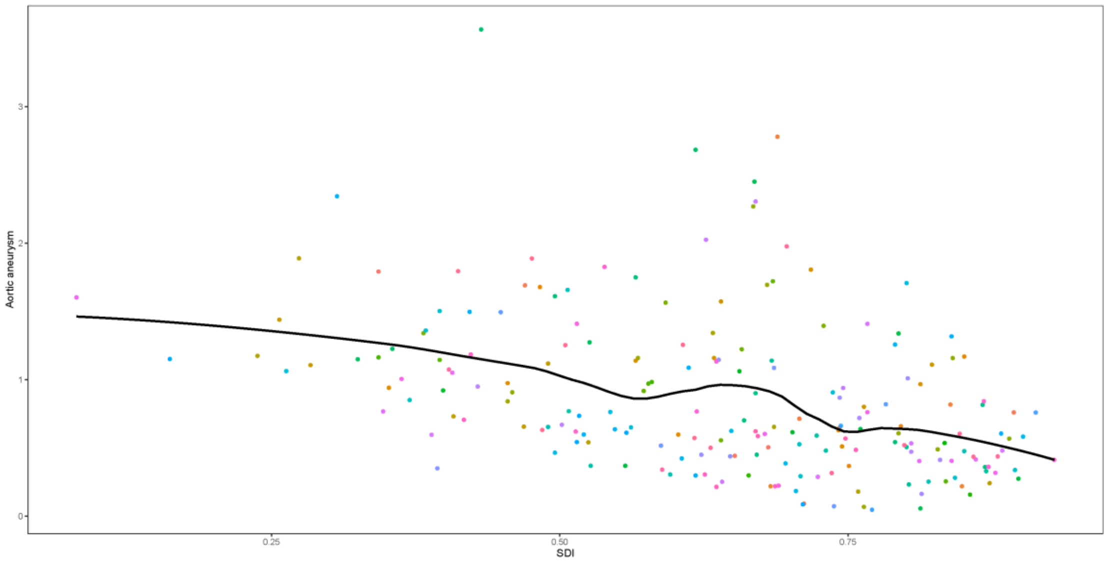

Aortic aneurysm
SDI
0
1
2
3
0.25
0.50
0.75

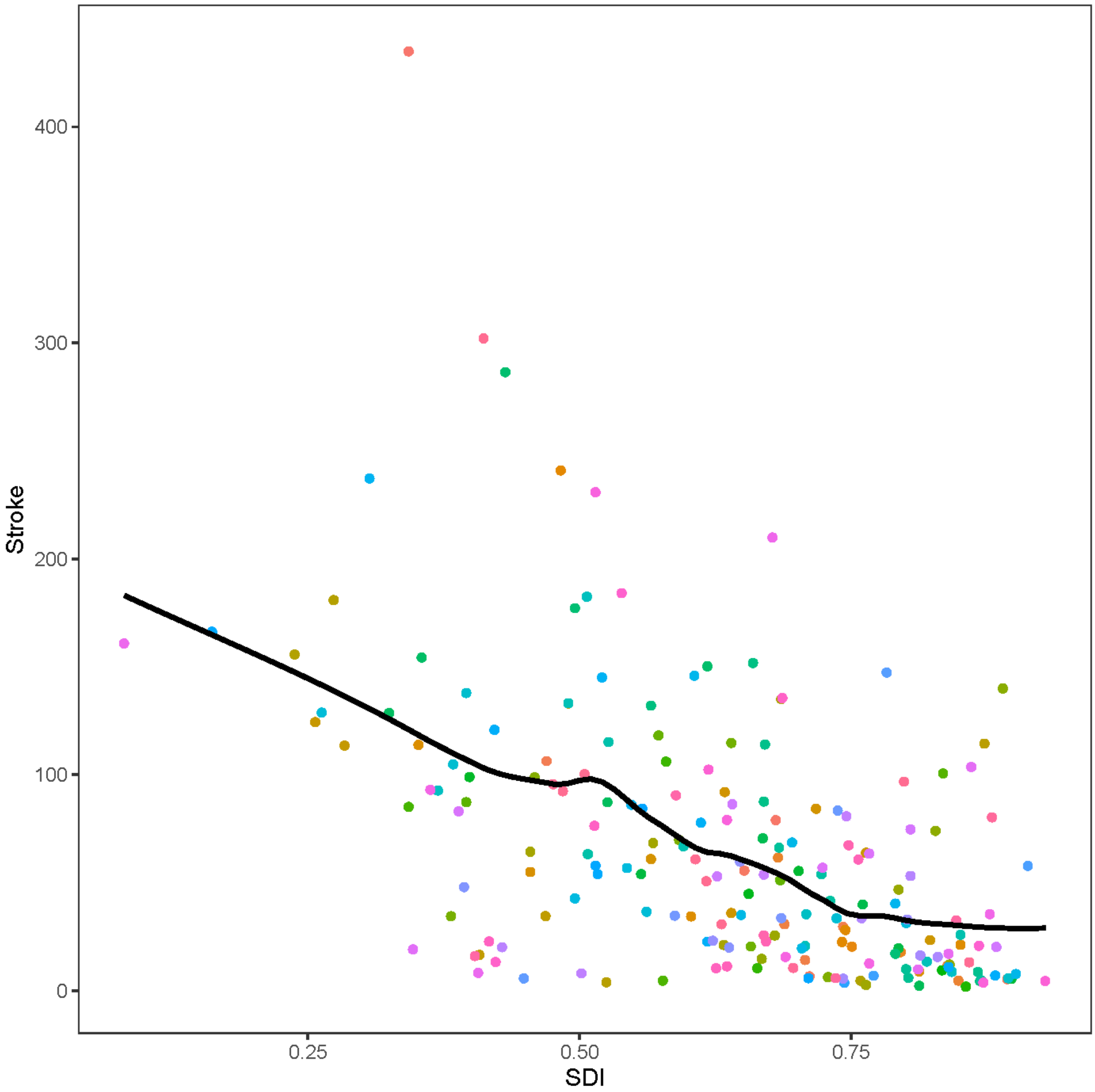

Stroke
SDI
0
100
200
300
400
0.25
0.50
0.75
